# BeeGUTS—A Toxicokinetic–Toxicodynamic Model for the Interpretation and Integration of Acute and Chronic Honey Bee Tests

**DOI:** 10.1002/etc.5423

**Published:** 2022-08-04

**Authors:** Jan Baas, Benoit Goussen, Mark Miles, Thomas G. Preuss, Ivo Roessink

**Affiliations:** ^1^ Wageningen Environmental Research Wageningen The Netherlands; ^2^ Ibacon Roßdorf Germany; ^3^ Bayer Monheim Germany

**Keywords:** Dose‐response modeling, Honey bee, Pesticides, Toxicokinetics, Toxicodynamics

## Abstract

Understanding the survival of honey bees after pesticide exposure is key for environmental risk assessment. Currently, effects on adult honey bees are assessed by Organisation for Economic Co‐operation and Development standardized guidelines, such as the acute and chronic oral exposure and acute contact exposure tests. The three different tests are interpreted individually, without consideration that the same compound is investigated in the same species, which should allow for an integrative assessment. In the present study we developed, calibrated, and validated a toxicokinetic–toxicodynamic model with 17 existing data sets on acute and chronic effects for honey bees. The model is based on the generalized unified threshold model for survival (GUTS), which is able to integrate the different exposure regimes, taking into account the physiology of the honey bee: the BeeGUTS model. The model is able to accurately describe the effects over time for all three exposure routes combined within one consistent framework. The model can also be used as a validity check for toxicity values used in honey bee risk assessment and to conduct effect assessments for real‐life exposure scenarios. This new integrative approach, moving from single‐point estimates of toxicity and exposure to a holistic link between exposure and effect, will allow for a higher confidence of honey bee toxicity assessment in the future. *Environ Toxicol Chem* 2022;41:2193–2201. © 2022 The Authors. *Environmental Toxicology and Chemistry* published by Wiley Periodicals LLC on behalf of SETAC.

## INTRODUCTION

With an ever‐increasing human population and its growing demand for crops that depend on insect pollination, the protection of this specific ecosystem service is receiving a lot of attention. Pollinators play an essential role in providing important pollination services to most wild plant species and cultivated crops. It is estimated that 78%–94% of crop species depend, at least to some extent, on animal pollination (Potts et al., 2016). These respective cropping systems, however, are also heavily dependent on the use of plant protection products. These products are by design highly effective against pest organisms but may involuntarily also impact pollinators. As a result, the use of plant protection products is highly regulated, and the registered compounds need to comply with a large variety of requirements to protect the user, the general population, and the environment. In the European Union, pesticides are regulated by regulation 1107/2009 (European Commission, 2009).

Although test methods for other species of pollinator are being developed, the standard test organism for regulatory testing has always been the honey bee (*Apis mellifera*). For this species, standard laboratory tests have been developed to evaluate potential effects of pesticide exposure of both adult bees and larvae. The present study focused on adult bees, and consequently the relevant laboratory tests comprised the acute contact (Organisation for Economic Co‐operation and Development [OECD] 214 [OECD, 1998b]), acute oral (OECD 213 [OECD, 1998a]), and chronic oral (OECD 245 [OECD, 2017]) toxicity to adult bees.

Acute tests usually last 48 h (although this can be extended to 96 h under certain specified conditions), whereas a chronic test lasts 10 days. The resulting LC50 (the exposure concentration which causes lethality to 50% of the exposed organisms at some specified point in time) or LD50 (the dose which causes lethality to 50% of the organisms at some specified point in time) is valid for the exposure pattern of the test and the time point for which it was derived. The different bee tests may lead to different conclusions on the toxicity of a compound, and typically the chronic tests lead to a more conservative result on the toxicity of the compound of interest. This is not because the compound is more toxic but because the test lasts longer and the incipient LD50 was not reached in the acute test (Heard et al., 2017). This raises questions because all tests are designed to measure the toxicity of a compound for bees, so an evaluation of the intrinsic toxicity of a compound should be independent of the test procedure.

Another drawback of current procedures is that extrapolating results to different exposure scenarios or different points in time is impossible (Ashauer et al., 2013). Even ranking the LD50 values for different compounds in terms of their toxicity needs to be carried out with great care because again the time dependency of the LD50 generally is not known and can lead to mismatches in toxic effects (Baas et al., 2010; Jager et al., 2006). A compound that has slow kinetics might be classified as not (very) toxic to bees in an acute test, but if effects were to be followed for a longer period of time, such a compound could be very toxic. An example of this for bee toxicity is cadmium, which in itself has a high toxicity to bees; but it takes time for the effect to develop because of its slow uptake kinetics (Heard et al., 2017).

These extrapolation and interpretation issues can be solved by using a mechanistic approach where time is explicitly taken into account and the effects are expressed in time‐independent parameters (Jager et al., 2006; Kooijman et al., 1996; OECD, 2006). Therefore, the aim of the present study was the development of a standard modeling framework for effects of chemicals on survival for adult honey bees. This framework allows incorporation of chronic and acute test results within one holistic modeling framework, moving away from the single‐point estimates that are currently used.

## MATERIALS AND METHODS

### Survival modeling framework

For the integration of acute and chronic tests, a modeling framework that integrates time‐dependent exposure patterns with time‐dependent effects is necessary. The best‐known and most suitable framework for modeling survival is the general unified threshold model of survival (GUTS). For a comprehensive description, including the mathematical details of the framework, see Ashauer et al. (2013, 2016), EFSA Panel on Plant Protection Products and Their Residues (PPR) et al. (2018), Jager & Ashauer (2018), and Jager et al. (2011). This toxicokinetic–toxicodynamic (TKTD) modeling framework was evaluated by the OECD and the European Food Safety Authority (EFSA) and recommended to be used in the evaluation of mortality data (EFSA PPR et al., 2018; OECD, 2006). The GUTS framework consists of two different survival models, the stochastic death (SD) model and the individual tolerance (IT) model. It is current practice to calculate parameter values for both assumptions and select either the more conservative or the one with the best fit (EFSA PPR et al., 2018).

The GUTS modeling framework can be used with external concentrations as a driving force for effects but also with internal concentrations as a driving force. The latter requires detailed knowledge on internal concentrations over time at the target site. Internal concentrations are generally not available for honey bees, and if they are available, it is mostly in the form of whole‐body residues (see Zaworra et al., 2019). The kinetics of internal organs, however, can be different from whole‐body residues, as was shown by Suchail et al. (2004) and Tada et al. (1987). Different physiological compartments of the bee, such as head, thorax, abdomen, hemolymph, midgut, and rectum, show very different kinetics. Therefore, there is no direct link between whole‐body residue kinetics and kinetics at the target site.

Because external concentrations are available and can be used as a driving force for effects, this was used as the starting point. This approach is generally referred to as the reduced GUTS model.

### Honey bee tests and implications for the modeling framework

#### Oral exposure

When a bee consumes food, it is first collected in the honey stomach, from which it can be taken up further or expelled (Fournier et al., 2014). The honey stomach is considered to be an inert vessel containing the pesticide, and therefore, the concentration of the pesticide in the honey stomach is the actual exposure concentration. The honey stomach therefore plays a crucial role in oral uptake, for both chronic tests and acute tests.

In an acute oral test (OECD, 1998a), the honey bees are fed contaminated food during an exposure period that typically lasts for 3–4 h. The bees are starved for up to 2 h prior to feeding them, to ensure that they will eat the contaminated food. It is assumed that the amount of pesticide in the honey stomach increases linearly during this exposure period.

**Figure 1:**
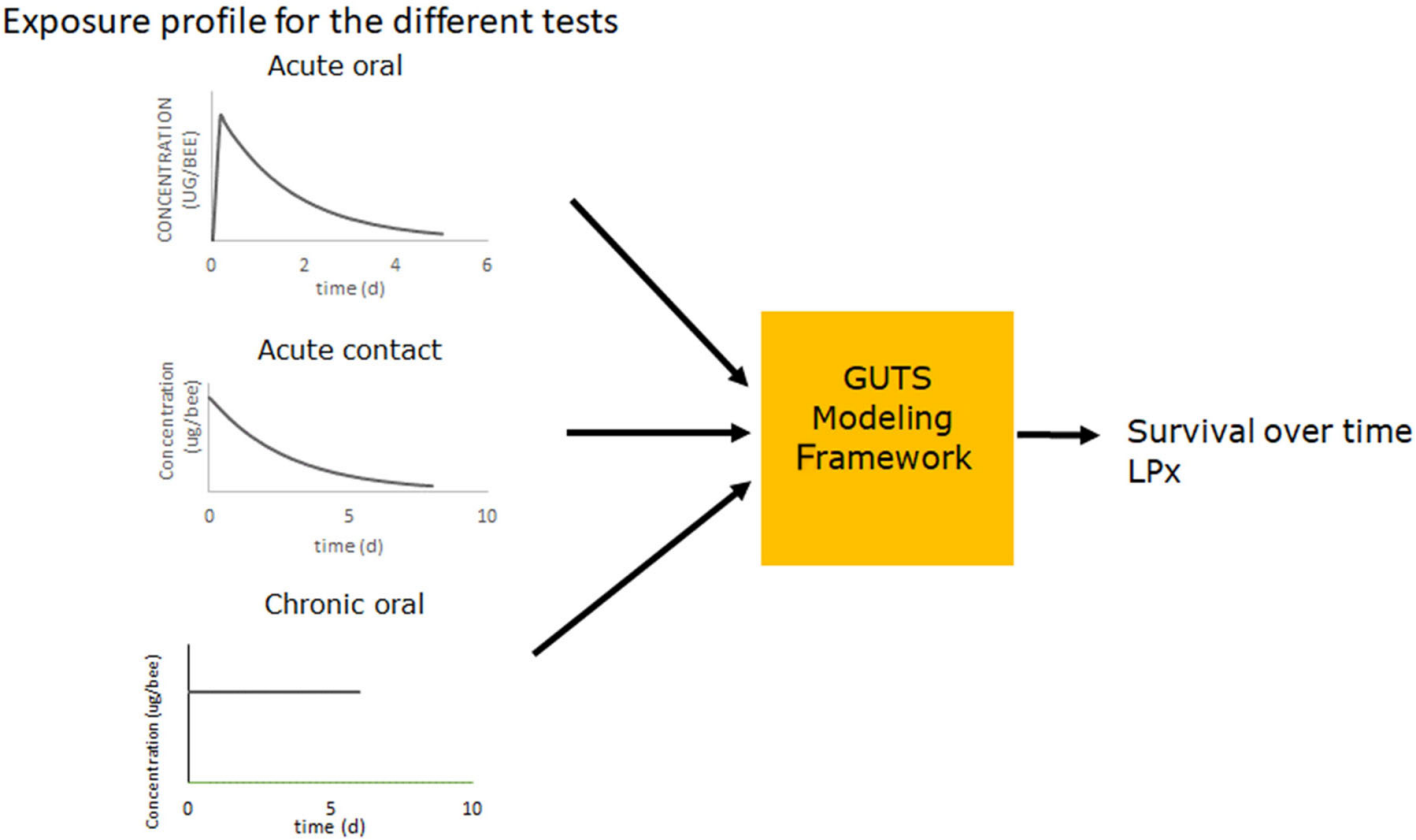
Model outline, with acute oral and chronic tests; the pesticide is taken up in the honey stomach, giving an effective concentration with a time‐dependent exposure profile. In an acute contact test, the pesticide is applied directly on the thorax of a bee, also leading to a time‐dependent effective concentration. The effective concentration feeds into the GUTS framework with survival over time and LP*x* values as output. GUTS = generalized unified threshold model for survival; LP*x* = the factor by which an entire exposure profile needs to be multiplied to yield *x*% lethality by the end of the exposure.

When the bees have consumed their contaminated food, the observation period starts, in which the bees are fed noncontaminated food ad libitum and effects are recorded at designated time points. The reported endpoint is the LD50 at the end of the test. In the observation period, the amount of pesticide in the honey stomach decreases over the observation period (see Figure 1). The decrease of the amount of pesticide in the honey stomach is a first‐order process which is determined by the volume of the honey stomach and the feeding rate, leading to the honey stomach release rate (*k*_sr_). Different values for the volume of the honey stomach can be found in the literature ranging from 30 to 50 µl (Becher et al., 2014; Visscher et al., 1996; Wolf et al., 1989); a default value was set at 40 µl. Feeding rates observed in the (chronic) tests are rather constant at approximately 25 µl/day. This gives a default value for the stomach release rate of 0.625 day^−1^ (see also the Supporting Information). The default value can be changed if experiment‐specific values are available.

In a chronic oral test (OECD, 2017), honey bees are continuously exposed to a constant concentration of a pesticide through their food during the 10‐day test period. The concentration in the honey stomach is considered to be constant (see Figure 1). The initial phase where the concentration in the honey stomach still builds up is considered to be negligible in the 10‐day test. This approach has been shown to give fits with *R* ^2^ values >0.9 of survival data over time for different compounds and different species of bees (Heard et al., 2017; Hesketh et al., 2016). The result of the test is a 10‐day LC50 value, but survival is recorded every 24 h after the start of the exposure (because food uptake is also recorded, the LC50 can be converted to an LD50).

#### Acute contact exposure

In an acute contact test (OECD, 1998b), the pesticide is applied as a 1–2‐µl droplet of a solution in a carrier solvent (e.g., acetone or weak solution of a commercial wetting agent for polar substances) on the dorsal thorax of the bee. Subsequently, the bees are put in cages and fed ad libitum with uncontaminated food, and effects are recorded at designated time points for the remainder of the observation period. The reported endpoint is the LD50 at the end of the test. The use of the carrier solvent ensures the uptake of the pesticide during the test period.

In general, a gradual decrease of the external concentration can be observed. Zaworra et al. (2019) measured whole‐body residue uptake and elimination kinetics for honey bees. In their study, three radiolabeled neonicotinoids (imidacloprid, acetamiprid, and thiacloprid) were administered with acetone as a carrier. The results showed that 58% (imidacloprid), 54% (acetamiprid), and 62% (thiacloprid) of the administered amounts were still present on the bee's cuticle after 24 h. These values are in line with the results obtained by Hillier et al. (2013), who measured residues on honey bees after dermal application of the miticides amitraz and tau‐fluvalinate. Their study showed that approximately 50% of tau‐fluvalinate and approximately 65% of amitraz were still present after 24 h. Other reported values in different species are 50% remaining residue in 24 h for a radiolabeled growth regulator from the benzoylphenylurea group in *Mamestra brassicae* (Tada et al., 1987) and a 75% decline in 24 h in topically applied pyrethrin I for the cockroach (Burt et al., 1971).

These data are remarkably similar for the different compounds tested, with very different physical and chemical properties, and even for different species. They show that the decrease of the concentration over time can be described with a first‐order contact availability rate constant (*k*_ca_). The default value of *k*_ca_ is set at 0.4 day^−1^ (see also Supporting Information, Section S2.2.2). This default value can be overruled if compound‐specific values are available. In bee testing a carrier solvent is used to enhance uptake of the pesticide over the bee cuticle; uptake without a carrier solvent is likely to be more compound‐ and species‐dependent, but the data are lacking.

#### Comparison of acute contact and acute oral exposure

The decline in concentrations for an acute contact and an acute oral test is rather similar in this approach. A very different argument to assume that the default values for the decrease of the exposure concentrations over time in acute oral and acute contact tests must be similar is the observation that the resulting 48‐h LD50s for acute contact and acute oral uptake are comparable (see Supporting Information) for some 40 different pesticides based on the results published by Sanchez‐Bayo and Goka (2016). If the observed effects in an acute oral test are comparable to those in acute contact test, this implies that also the exposure pattern must be comparable.

### General outline of the model

The general outline of the BeeGUTS model is presented in Figure 1.

### Honey bee test data

Raw test results were made available by Bayer within the framework of their transparency commitments for acute oral, acute contact, and chronic tests; and reported LD50s or LC50s for chronic tests are shown in Table 1. All tests were carried out under good laboratory practices and according to the appropriate OECD guidelines mentioned in the introduction (OECD, 1998a, 1998b, 2017) or, if they predate the OECD guidelines, according to similar procedures.

**Table 1:** Overview of test results for the different compounds: Each line summarizes the result of a single test

Compound	Bayer report no.	48‐h LD50 acute oral (µg/bee)	48‐h LD50 acute contact (µg/bee)	10‐day LC50 chronic (mg/kg)
Aclonifen	M‐601664	>107	100	>90
Aclonifen	M‐174936	–	–	>3000
Beta‐cyfluthrin	M‐051896	0.13	0.006	–
Beta‐cyfluthrin	M‐363013	0.016	0.038	–
Beta‐cyfluthrin	M‐053813	0.050	0.012	–
Beta‐cyfluthrin	M‐479053	–	–	0.019 µg/bee
Bromoxynil	M‐483226	–	–	350
Bromoxynil	M‐451407	>201	>201	–
Bromoxynil	M‐444560	10.8	>201	–
Deltamethrin	M‐149494	–	0.28	–
Deltamethrin	M‐149496	1.41	–	–
Deltamethrin	M‐444971	0.2	0.12	–
Deltamethrin	M‐477250	–	–	15.1
Ethiprole	M‐192387	0.034	0.013	–
Ethiprole	M‐214951	0.033	0.057	–
Ethiprole	M‐581904	–	–	46
Fenitrothion	M‐293568	0.50	0.48	–
Fenamidone	M‐191659	85 (72 h)	>160	–
Fenamidone	M‐421624	57 (72 h)	>93	–
Fenamidone	M‐470658	–	–	86
Fenoxaprop	M‐577004	–	–	420
Fenoxaprop	M‐470702	>109	>100	–
Imidacloprid	M‐600686	–	–	1.31
Imidacloprid	M‐006940	0.0037	0.081	–
Imidacloprid	M‐016942	0.0409	–	–
Imidacloprid	M‐067751	>0.0347	0.0429	–
Imidacloprid	M‐067996	>0.045	–	–
Imidacloprid	M‐068023	>0.0703	0.0749	–
Methiocarb	M‐357085	0.44	0.11	–
Methiocarb	M‐013166	0.8	0.43	–
Metribuzin	M‐540903	–	–	620
Metribuzin	M‐014115	>166	>200	–
Metribuzin	M‐294086	34	>100	–
Spiromesifen	M‐657628	–	–	9.47
Spiromesifen	M‐031874	792	>200	–
Spiromesifen	M‐030406	60	>200	–
Spirotetramat	M‐298419	>109	>100	–
Spirotetramat	M‐081227	>107	>100	–
Spirotetramat	M‐395773	>111	>100	–
Spirotetramat	M‐572046	–	–	26
Tebuconazole	M‐105205	42	>200	–
Tebuconazole	M‐182469	182	302	–
Tebuconazole	M‐103501	910	>4000	–
Thiacloprid	M‐000856	17.3	38.8	–
Thiacloprid	M‐001004	12.8	51.6	–
Thiacloprid	M‐475374	–	–	50.9
Tetraniliprole	M‐438810	0.11	0.97	–
Tetraniliprole	M‐441758	0.01	1.2	–
Tetraniliprole	M‐551955	–	–	0.58

LD50 = median lethal dose; LC50 = median lethal concentration.

In addition to these raw test data, we used literature data on a chronic test for dimethoate and imidacloprid (Department for Environment, Food and Rural Affairs, 2007).

All chronic studies had 10 observations over time on mortality ranging from no effects to strong effects (>90% effect on mortality in the highest concentration at 10 days; see Supporting Information, Section S3), with the exception of aclonifen which showed no effects up to the highest concentration, resulting in a reported 10‐day LC50 >90 mg/kg. The acute test results generally have observations at three to five points in time depending on if there is a prolongation of the test. The first observation on mortality is after 4 h of exposure, which is often too soon to observe any effects.

Compounds were selected for further analysis if a chronic oral, an acute oral, and an acute contact test were available (which is considered a complete data set). If a chronic test was missing or not carried out as a dose–response test (e.g., limit test), the data set for this compound was considered to be incomplete and therefore not considered for further analysis. For some compounds more than one acute oral or acute contact test were available. If the results of these tests showed a variation in excess of a factor of 10, the data set for such a compound was considered to be inconsistent or beyond the range of reasonable experimental variability and treated separately.

The experimental variability can be estimated for the positive control (dimethoate; see Table 2). The result of the positive control should be within 0.10–0.35 µg/bee for an acute oral test and 0.10–0.30 µg/bee for an acute contact test for a test to be valid.

**Table 2:** Summary of the acute test results for dimethoate

Metric	Acute oral LD50 (µg/bee)	Acute contact LD50 (µg/bee)
Number of studies	22	22
Average LD50	0.161	0.198
Median LD50	0.13	0.195
Standard deviation	0.073	0.059
Maximum value	0.35	0.34
Minimum value	0.081	0.09

LD50 = median lethal dose.

The range in the results for dimethoate for all available tests is well within a factor of 5 (slightly higher than based on the guidelines because one acute contact test and one acute oral test had results lower than described, but the tests were still accepted because the bees were more sensitive than might be expected). It is to be expected that the range in the results for the test compounds will be larger because they can be more difficult to handle than dimethoate; hence, the factor of 10 was chosen to assign inconsistency to a data set.

Overall, this gave five complete and consistent data sets: beta‐cyfluthrin, dimethoate, deltamethrin, ethiprole, and thiacloprid. Seven compounds had complete data sets but with differences greater than a factor of 10 in reported LD50s for the same test: bromoxynil, fenamidone, fenoxaprop, imidacloprid, metribuzin, spiromesifen, and tetranilliprole. The data sets for aclonifen, fenitrothion, methiocarb, spirotetramat, and tebuconazole were incomplete.

### Calibration, validation, and application of the model

The EFSA Scientific Opinion on TKTD modeling (EFSA PPR et al., 2018) outlines the requirements for the available data for calibration and validation of the model for aquatic organisms. For model calibration, at least five points in time are needed. For model validation, time‐dependent survival data (preferably with a pulsed exposure) which have not been used for model calibration are to be used. For application of the model to bee survival, the model is calibrated with the chronic data sets (10 observations over time) and validated with the acute data. This does not fully comply with the EFSA guidelines because the validation data do not have at least seven observations over time and a repeated pulse experiment was not available for any of the compounds. However, two time‐dependent data sets were available, based on different exposure routes; therefore, the validation is considered to be compliant with the EFSA guidelines. The time dependency of the validation data set is considered to be the most important requirement.

### Model calibration

In Table 3, an overview of the model performance for calibration is presented. The evaluation criteria used were based on the EFSA Scientific Opinion (EFSA PPR et al., 2018): 1) the quality of the visual fit, which is a subjective expert opinion on the quality of the model fit to the data. 2) The normalized root mean square error, which is a criterion for the match between the model output and the survival data over all time points (a value <30% is considered to be good). 3) The survival probability prediction error, which compares the predicted and observed survival at the end of the experiment (a value <50% is considered to be good; a negative value indicates an underestimation of effects, and a positive value indicates an overestimation).

**Table 3:** Overview of the available chronic test results with the goodness‐of‐fit criteria based on the European Food Safety Authority scientific opinion on toxicokinetic–toxicodynamic models

Compound	SD/IT model	Visual fit	NMRSE (%)	SPPE min. (%)	SPPE max. (%)
Beta‐cyfluthrin	SD	Good	7.58	−4.11	6.34
	IT	Good	6.77	−4.42	4.90
Bromoxynil	SD	Good	6.63	−6.80	10.20
	IT	Good	3.96	−7.23	6.28
Deltamethrin	SD	Fair	9.96	−3.32	18.10
	IT	Poor	12.87	−12.80	9.47
Dimethoate	SD	Good	10.07	−11.10	10.00
	IT	Good	10.34	−7.69	6.62
Ethiprole	SD	Fair	4.55	−9.82	13.30
	IT	Good	2.89	−7.87	2.40
Fenamidone	SD	Good	5.18	−31.20	7.41
	IT	Fair	7.67	−39.50	4.56
Fenoxaprop	SD	Fair	12.02	−7.14	10.80
	IT	Fair	10.57	−12.60	4.76
Imidacloprid	SD	Poor	6.83	−6.31	16.70
	IT	Fair	3.54	−5.81	8.86
Metribuzin	SD	Good	5.05	−2.35	2.92
	IT	Good	3.41	−3.45	0.65
Spiromesifen	SD	Good	3.89	−7.08	6.23
	IT	Good	4.59	−6.73	5.41
Spirotetramat	SD	Fair	9.82	−18.90	0.65
	IT	Poor	14.20	−35.60	0.34
Tetraniliprole	SD	Good	1.87	−1.74	1.59
	IT	Good	2.92	−2.83	4.45
Thiacloprid	SD	Good	5.44	−9.09	2.37
	IT	Good	5.24	−13.30	7.58

SD = stochastic death; IT = individual tolerance; NMRSE = normalized root mean square error; SPPE = survival probability prediction error.

The last criterion mentioned in the EFSA Scientific Opinion, the posterior predictive check, was not calculated in our case because the statistical framework to calculate this in the open GUTS framework is currently not defined.

Table 3 shows that the overall goodness‐of‐fit values and visual interpretation match the criteria laid out in the EFSA Scientific Opinion (EFSA PPR et al., 2018).

### Model validation

The calibrated model output is used for validation with the available time‐variable exposures. The five complete and consistent data sets were used. All validation data sets met the requirements from the EFSA Scientific Opinion (see Supporting Information).

### Model application

With the validated model, all available test data were combined; and subsequently, the model was recalibrated to show that both acute and chronic test results can be interpreted with one consistent modeling framework, leading to one set of parameter values that describe the toxicity of a compound.

## RESULTS AND DISCUSSION

### Integration of chronic and acute tests for complete and consistent data sets

**Figure 2:**
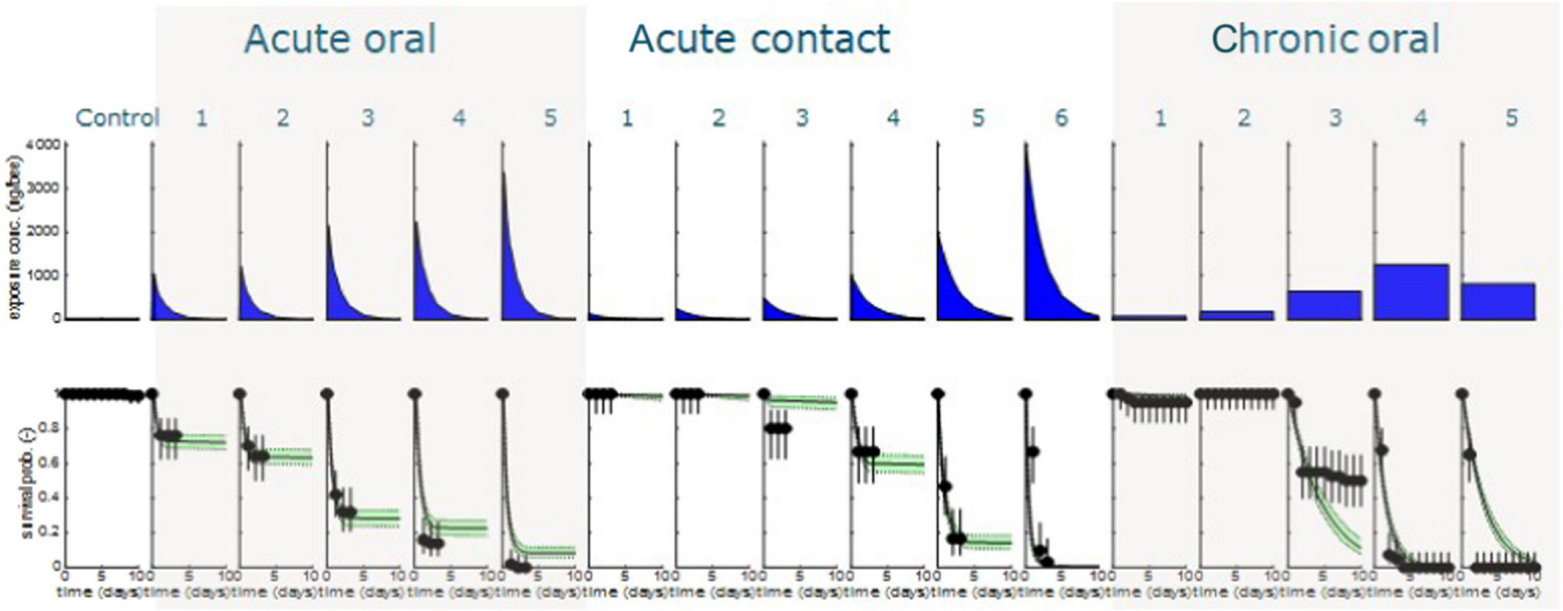
Deltamethrin control, five acute oral treatments, six acute contact treatments, and the five chronic treatments with a 10‐day observation period. Top panels give the exposure profile and bottom panels, the fitted survival probability (line with green confidence intervals) against the observed survival data (dots).

**Figure 3:**
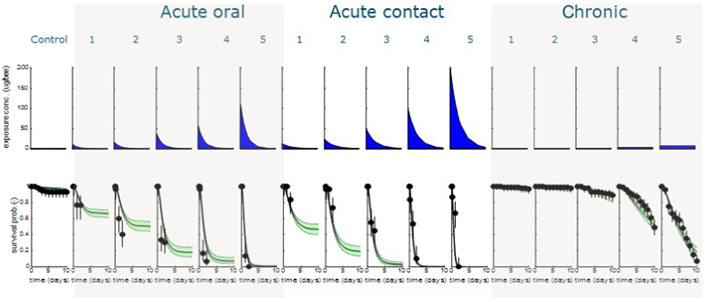
Thiacloprid control, five acute oral treatments, five acute contact treatments, and the five chronic treatments with a 10‐day observation period. Top panels give the exposure profile and bottom panels, the fitted survival probability (line with green confidence intervals) against the observed survival data (dots).

Complete and consistent data sets were analyzed with the BeeGUTS model, taking into account the specifics of the procedures in a bee toxicity test and bee physiology. As an example, the results of the SD model are presented for deltamethrin (Figure 2) and thiacloprid (Figure 3). The plots for the other compounds for both the SD and IT models are shown in the Supporting Information.

The figures, see also the Supporting Information, show that all tests can be integrated for all compounds with a complete and consistent data set with the default settings for *k*_ca_ and *k*_sr_. The goodness‐of‐fit criteria for all compounds are well within the thresholds given by EFSA (see Supporting Information). This leads to an integrated consistent evaluation of the toxic effects with one set of parameters that describe the toxic effects.

### Integration of chronic and acute tests for complete but inconsistent data sets

For bromoxynil, fenamidone, fenoxaprop, imidacloprid, metribuzin, spiromesifen, and tetraniliprole, at least two acute tests are available (either contact or oral) with a difference in reported LD50s of at least a factor of 10. In this case, it is possible to check if any of the available test results fits with the chronic test or if there is another reason for the differences. Thus, an important use of the model lies in the possibility of verification of test results and a check on a consistent or time‐dependent mechanism of toxicity.

The figures (see also the Supporting Information) show that for all the inconsistent data sets it is possible to identify the acute test that does not fit the chronic data as an outlier, except for spiromesifen. Spiromesifen belongs to the chemical class of keto‐enols (subclass tetramic acid derivatives), which disrupt lipogenesis in insects via the inhibition of acetyl coenzyme A (CoA) carboxylase (Sparks & Nauen, 2015). In the short term, this effect is not lethal; and in acute tests, this compound is indeed not very toxic compared to a chronic test. Despite a high discrepancy in the outcome of the two oral endpoints (acute contact test showed a 48‐h LD50 of >200 µg/bee, and the two acute oral tests showed 48‐h LD50s of 60 and 792 µg/bee, respectively). In a chronic test, however, prolonged continuous inhibition of acetyl CoA carboxylase will occur; and, as a consequence, the bees will starve to death. In the chronic test the bees started to show effects after 3–4 days of exposure at a dose of 0.6 µg/bee. In this case the acute test does not reflect the actual toxicity of such compounds. However, the time‐dependent working mechanism can be shown with the model by a lack of possible integration of acute and chronic effects.

### Implications of the model and outlook

A novel method was developed and successfully validated for the interpretation of toxicity tests for honey bees, based on a large and diverse data set. The model allows for a better interpretation/prediction of effects of field‐realistic exposures, comparison of sensitivity of different bee species, and a realistic evaluation of simultaneous exposure to different pesticides.

#### Evaluation of effects under field‐realistic exposures

When the parameters describing the effects are derived from tests carried out in a laboratory, these values can be used to evaluate effects (or calculate LD50s) for complex exposure patterns for any point in time (Ashauer et al., 2016; Pieters et al., 2006). Therefore, they allow for a much more realistic evaluation of effects under field‐realistic conditions with time‐variable exposures. Effects can be calculated for oral and contact exposure separately and later combined by adding their scaled damage and, as such, treating them as a simultaneous exposure to two compounds acting on an identical target (Bart et al., 2021).

Effects for oral uptake can be evaluated without any further assumptions. For contact exposure this is more difficult because it is not known if contact uptake without a carrier solvent will lead to similar results as exposure in an acute test. However, in a first‐tier assessment effects of contact exposure can be calculated with the assumption that the external concentration experienced by the bee is the driving force for effects, therefore using the default parameters given in the model description. This will give a maximum effect that can be expected for a contact exposure and, as such, is suitable for an effect assessment under real‐life exposure conditions, something that is not possible with the current approaches based on LD50s. In addition, a standard feature of the GUTS model is the possibility to calculate safety margins (expressed as LP10 or LP50, the factor by which an entire exposure profile needs to be multiplied to yield 10% or 50% lethality by the end of the exposure).

#### Sensitivity of bees to pesticides

A consequence of the use of the BeeGUTS model is a different interpretation of the LD50 in an acute test. The acute oral test is designed to test acute toxicity effects to honey bees after a single application or dosing event. In contrast to an acute aquatic test for fish or daphnia, exposure is not constant over time. The different exposure regimes in the various bee tests do not allow for a one‐to‐one comparison of the resulting LD50 values. Current practice, based on the EFSA guidelines (EFSA PPR, 2012), is to derive the LD50 from both the acute oral and contact tests to the dose that is administered at *t* = 0 days. Therefore, if we want to compare the different test results (including the chronic test), we need to know the actual exposure profile and recalculate the acute test to constant exposure or the chronic test to a single dose. Which one is relevant depends on the question addressed by the risk assessment. If exposure is given not as a time series but at time point 0, calculating the single dose would be more appropriate for a field situation.

With the integrated approach a very different and more realistic proxy for the sensitivity of the bees is obtained: the effect threshold as a parameter of the model (Baas & Kooijman, 2015). The effect threshold is by definition the LD0 for infinite exposure time, which is not dependent on time and, with this approach, also independent of a test. Generally, this value will be significantly lower than the LD50 in an acute test and lower than the LD50 that can be derived from a chronic test.

This effect threshold can, with the appropriate data, also be generated for other bee species, including non‐*Apis* bee species, allowing for a real comparison of their toxicity for different compounds. This type of comparison is currently based on acute tests (most frequently acute contact tests), but the exposure profile is likely to be different for different species, depending on the physiology and morphology of the bee species (e.g., body hair, cuticle thickness, and body size). This has not been considered up until now and might lead to different conclusions on sensitivity of different bee species from an evaluation based on 48‐h LD50s.

#### Effects of simultaneous exposure to multiple compounds

Effects of simultaneous exposure to multiple compounds can also be taken into account with the BeeGUTS modeling framework. In a first assessment, synergistic or antagonistic effects can be neglected. Then we have two principal possibilities: The compounds do not share a common target site, or the compounds do share a common target site. In the first case the survival probabilities for each compound to which a bee is exposed can be multiplied to obtain the combined effect of the total exposure (Baas et al., 2007). In the second case, the scaled damage can be added, and the survival probability can then be calculated for the combined effect of the total exposure (Bart et al., 2021).

## Supporting Information

The Supporting Information is available on the Wiley OnlineLibrary at https://doi.org/10.1002/etc.5423.

## Author Contributions Statement

**Jan Baas**: Methodology; Formal analysis; Validation; Writing—original draft. **Benoit Goussen**: Methodology; Visualization; Validation; Writing—review & editing. **Mark Miles**: Data curation; Writing—review & editing. **Thomas Preuss**: Conceptualization; Methodology; Supervision; Visualisation; Writing—review & editing. **Ivo Roessink**: Conceptualization; Supervision; Funding acquisition; Project administration; Writing—review & editing.

## Supporting information

This article includes online‐only Supporting Information.

Supporting information.

## Data Availability

As part of the transparency policy of Bayer Crop Sciences the full reports can be requested by sending an email to cropscience-transparency@bayer.com referring to the present study and the report numbers of interest.
