# Supplementary material for: BeeGUTS—A Toxicokinetic–Toxicodynamic Model for the Interpretation and Integration of Acute and Chronic Honey Bee Tests
Source: Environ Toxicol Chem. 2022 Aug 4;41(9):2193–201. doi: 10.1002/etc.5423 (PMC9541331; doi:10.1002/etc.5423)
Supplement: Supplementary file 1 — Supporting information. [file ETC-41-2193-s001.docx]

Supporting Information

BeeGUTS – A TKTD model for the interpretation and integration of acute and chronic honey bee tests

Jan Baas^1^, Benoit Goussen ^2^, Mark Miles ^3^, Thomas G. Preuss ^3^, Ivo Roessink ^1^

^1^Wageningen University and Research, Droevendaalsesteeg 4, 6708 PB Wageningen

^2^ibacon GmbH, Arheilger Weg 17, 64380 Roßdorf, Germany

^3^BAYER, Alfred-Nobel Str. 50, 40789 Monheim, Germany

Contents

[1 Formal model 3](#_Toc85808191)

[1.1 Acute oral exposure 3](#_Toc85808192)

[1.2 Acute contact exposure 3](#_Toc85808193)

[1.3 Chronic exposure 3](#_Toc85808194)

[2 MODEL EVALUATION 5](#_Toc85808195)

[2.1 Model Evaluation 5](#_Toc85808196)

[2.2 Model sensitivity analysis of the exposure profiles 6](#_Toc85808197)

[2.2.1 Honey stomach release rate 6](#_Toc85808198)

[2.2.2 Contact uptake availability rate constant 10](#_Toc85808199)

[2.2.3 Comparison between acute oral and acute contact test results 15](#_Toc85808200)

[3 Model Validation 16](#_Toc85808201)

[3.1 Beta-cyfluthrin 16](#_Toc85808202)

[3.2 Deltamethrin 17](#_Toc85808203)

[3.3 Dimethoate 19](#_Toc85808204)

[3.4 Ethiprole 20](#_Toc85808205)

[3.5 Thiacloprid 22](#_Toc85808206)

[3.6 Conclusion on validation 23](#_Toc85808207)

[4 Fits for compounds with consistent and complete datasets 25](#_Toc85808208)

[4.1 Beta-cyfluthrin 25](#_Toc85808209)

[4.1.2 SD model fits 25](#_Toc85808210)

[4.1.2 IT model fits 27](#_Toc85808211)

[4.2 Deltamethrin 30](#_Toc85808212)

[4.2.1 SD model fits 30](#_Toc85808213)

[4.2.2 IT model fits 33](#_Toc85808214)

[4.3 Dimethoate 36](#_Toc85808215)

[4.3.1 SD model fits 36](#_Toc85808216)

[4.3.2 IT model fits 38](#_Toc85808217)

[4.4 Ethiprole 39](#_Toc85808218)

[4.4.1 SD model fits 39](#_Toc85808219)

[4.4.2 IT model fits 41](#_Toc85808220)

[4.5 Thiacloprid 42](#_Toc85808221)

[4.5.1 SD model fits 42](#_Toc85808222)

[4.5.2 IT model fits 44](#_Toc85808223)

[5 Compounds with inconsistent and complete datasets 47](#_Toc85808224)

[5.1 Bromoxynil 47](#_Toc85808225)

[5.1.1 SD model 47](#_Toc85808226)

[5.2 Fenamidone 48](#_Toc85808227)

[5.3 Fenoxaprop 48](#_Toc85808228)

[5.4 Imidacloprid 48](#_Toc85808229)

[5.5 Metribuzin 49](#_Toc85808230)

[5.6 Spiromesifen 49](#_Toc85808231)

[5.7 Tetraniliprole 50](#_Toc85808232)

[6 References 52](#_Toc85808233)

# 1 Formal model

The BeeGUTS model is based on the reduced GUTS framework (Jager 2011; Ashauer 2016) with additional assumptions linked to the uptake of compound. These additional assumptions are only focusing on refining the exposure profile over time, i.e. the dose at which the bees are really exposed over the course of the experiment, accounting for the bee physiology. The other assumptions and parameters of the reduced GUTS framework are conserved. The reduced GUTS model is already well documented ^1-4^. As such, the current section focuses on the additional assumptions.

The main additional assumption is that the uptake of compound is modulated based on the physiology of the bee and the route and type of exposure. Two exposure routes were considered - oral and contact, as well as two exposition types – acute and chronic. Each exposure route leads to a specific exposure profile, with a time dependent exposure concentration (*C_t_ (t)* in equations 2, 5, and 6), which is the input for the GUTS model

In the standard assessment of an acute test for instance, a constant exposure is assumed over the 48 hours observation period. The reported LD50s, usually in µg bee^-1^ are based on this assumption. However, the exposure declines over time due to the bee physiology.

Standard oral tests usually provide an exposure dose in µg bee^-1^. This accounts for the exposure concentration in the food and the bee feeding rate. When this was not the case and the exposure provided is based on the food concentration, the exposure dose per bee can be recalculated using

| $C_{exp}=\frac{f_{eedrate}}{1000}C_{food}$ | (1) |
| --- | --- |

With $C_{exp}$ (µg bee^-1^) the exposure dose by bee based on the experimental setup only, $f_{eedrate}$ (mg feed bee^-1^), the feeding rate provided in the experimental data (0.25 mg d^-1^ as a default if not provided), and $C_{food}$ (mg kg^-1^ feed) the concentration of active substance in the food.

## 1.1 Acute oral exposure

In an acute oral exposure test, a bee is starved for a short period and subsequently fed the compound of interest in cohorts of 10 individual bees. During this period the honey stomach is filled with food containing the compound of interest. This exposure period typically lasts a few hours after which the bees are put in cages and from there on they are fed non-contaminated food and the observation period starts. The observation period lasts at least 2 days but can be extended based on the development of effects. Considering the honey stomach as an inert reservoir from which the bee feeds itself, the most physiologically relevant representation of the exposure scenario was obtained by using a constant increase of the concentration during the exposure period followed by a first order decline (Equation 2):

| $C_{t}(t)=\left( C_{exp}\left( \frac{t}{t_{exp}} \right)+C_{exp}e^{-k_{sr}\left( t-t_{exp} \right)} \right)\text{ with}$ $C_{exp}\left( \frac{t}{t_{exp}} \right)\text{ = 0 for }{t>t}_{exp}$ $C_{exp}e^{-k_{sr}(t-t_{exp})}\text{= 0 for }{t\leq t}_{exp}$ | (2)  (3)  (4) |
| --- | --- |

With $C_{t}$ *(t)* (µg bee^-1^) the effective concentration, i.e. the dose at which the bees are really exposed over the course of the experiment accounting for their physiology, $C_{exp}$ (µg bee^-1^) the constant exposure dose provided by the experimental setup, $t_{exp}$ (days) the exposure period, $t$ (days) the time, and $k_{sr}$ (d^-1^) the stomach release rate.

## 1.2 Acute contact exposure

In an acute contact exposure test, a droplet of the compound is usually combined with a carrier compound and put on the ventral thorax of the bee. The carrier compound, usually acetone or DMSO), increases the uptake; inducing a more or less instantaneous start of the exposure. The compound concentration then decreases with time due to the uptake and breakdown processes. In this context, the exposure scenario was obtained using a first order decline similar to the oral exposure one:

| $C_{t}(t)=C_{exp}e^{-k_{ca}t}$ | (5) |
| --- | --- |

With $C_{t}$ *(t)* (µg bee^-1^) the effective concentration, i.e. the dose at which the bees are really exposed over the course of the experiment accounting for their physiology, $C_{exp}$ (µg bee^-1^) the constant exposure dose provided by the experimental setup, $t$ (days) the time, and $k_{ca}$ (d^-1^) the contact availability rate.

In the absence of a carrier, the uptake will be slower and is not covered by the current equation.

## 1.3 Chronic exposure

In the case of a chronic exposure test, the exposure is assumed constant during the whole test period. In reality of course the exposure profile will start at a concentration of 0 and increase to the concentration in the food. How fast the build-up of the concentration will take place will depend on feeding events, but reliable data on this are missing so we take a conservative approach and assume a constant exposure over the entire observation period of 10 days. Possible observed concentration spikes will be flattened by the presence of the honey stomach which acts as an inert reservoir. As such, the exposure scenario is considered constant and identical to the experimental exposure concentration:

| $C_{t}=C_{exp}$ | (6) |
| --- | --- |

With $C_{t}$ (µg bee^-1^) the effective concentration, i.e. the dose at which the bees are really exposed over the course of the experiment accounting for their physiology and $C_{exp}$ (µg bee^-1^) the constant exposure dose provided by the experimental setup

# 2 MODEL EVALUATION

The modelling framework is based on the General Unified Threshold model of Survival or GUTS. See ref ^1-5^ for a comprehensive description, including the mathematical details of the framework. This Toxico-kinetic Toxico-dynamic (TKTD) modelling framework was evaluated by EFSA and the OECD and recommended to be used in the evaluation of mortality data ^1, 6^. The GUTS framework consists of the two different survival models, the stochastic death model (SD) and the individual tolerance model (IT). It is current practice to calculate parameter values for both assumptions and select either the conservative one or the one which fits best ^1^.

Since we have only external data available (see section 1 of this Supporting Information) these were chosen as the driving force for effects, so the reduced GUTS model was used as the heart of the modelling framework. This was modified to capture the specifics of different honey bee tests and the physiology of the honey bee, resulting in the BeeGUTS framework. The general assumption is that with oral uptake the pesticide is taken up in the honey stomach, which acts as an inert reservoir from which the pesticide is taken up over time ^7^. With contact uptake a small droplet of a solution containing the pesticide is dropped in the neck of the bee and then uptake starts ^8^. The general outline of the model is presented in Figure 1.


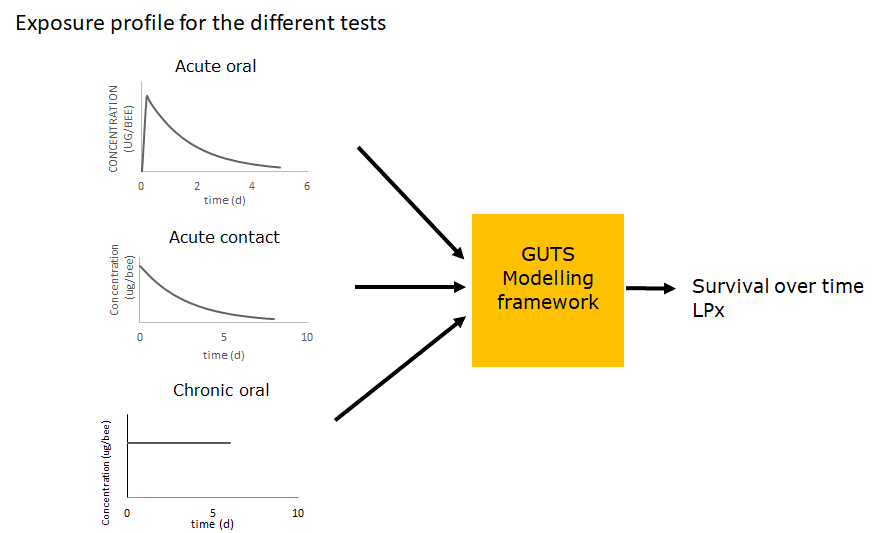


***Figure 1****: Model outline; Different exposure routes each with their specific effective concentration profile is input for the GUTS model and is the driving force for effects. The output is the parameter values that determine the survival over time and the margin of safety in the form of LPx values*

The model evaluation and validation is based on the EFSA Scientific opinion (EFSA SO)^1^. The recommendations described in this document are followed for this model and parts of the text of the EFSA SO is used here.

## 2.1 Model Evaluation

Model parameters are estimated for a specific combination of species and compound. Supporting data for GUTS models are mortality or immobility data, see section 1 of this SI. Sensitivity analysis quantifies the influence of parameters on the model outputs. For the reduced GUTS models, the influence of the model parameters on the model results is described in the EFSA SO and does not need to be reported on a case-by-case basis.

The reduced GUTS model is well documented ^1-4^ for fish and invertebrates and set up according to the EFSA Scientific opinion on TKTD modelling. This implies that the conceptual model, the formal model, the computer model require no further model validation. An executable version of the model is available in the form of the Openguts software, either as a standalone version or as a MATLAB software package.

## 2.2 Model sensitivity analysis of the exposure profiles

The sensitivity analysis focusses on the exposure profiles that serve as input in the reduced GUTS model.

### 2.2.1 Honey stomach release rate

The honey stomach release rate (*k_sr_*) is determined by the volume of the honey stomach and the feeding rate. Typical volumes for the honey stomach found in literature are between 30 and 50 µL. Here it has to be noted that the honey stomach does not need to be at its full capacity. When an acute oral test starts the honey bees are starved for some 2 hours and after that they are fed the contaminated food in the exposure period, which typically lasts between 2 and 6 hours where each bee consumes 20 µL of food ^9^. After that the observation period starts where the bees are fed ad-libitum non-contaminated food at a typical feeding rate of 25 µL/day.

The lowest value for the volume of the honey stomach would be 20 µL (the initial feeding volume) and the highest value would be 50 µL. Probably the honey stomach is not completely empty when the bees are fed the contaminated food, therefore a default setting of the honey bee stomach volume was set at 40 µL/day. From the start of the observation period there is no further uptake of the pesticide and therefore a first order decay of the amount of pesticide in the honey stomach is assumed, governed by the volume of the honey stomach and the feeding rate. The underlying implicit assumption is that the honey stomach acts as an inert vessel from which the bee takes up the pesticide; or in other words the honey stomach is treated as an external reservoir from which the pesticide is taken up by the bee. In addition it is assumed that during the exposure phase there is negligible decline due to dilution (or uptake) of the amount in the honey stomach.

The minimum and maximum volume for the honey stomach give *k_sr_* values of 1.25 and 0.5 d^-1^ resp (based on the typical observed feeding rate of 25 µL/day) and the default setting is 0.625 d^-1^. This gives an exposure profile as is shown in figure 2.

*Figure 2, Exposure profile for an acute oral test. In the first phase (typically lasting 2 hours) a linear increase of the amount of pesticide in the honey stomach is assumed, followed by a first order decay governed by the feeding rate and the honey stomach volume.*

The maximum, minimum and default value for *k_sr_* were used to investigate the sensitivity of the model for this parameter. Thiacloprid is chosen as a trial compound as Thiacloprid has a consistent dataset with good fits of the calibration dataset and NMRSE and SPPE values well within acceptable boundaries (see the main text).

The range of values for the honey stomach release rate were used for integration of acute and chronic data and evaluated, both with the SD model (figures 3-5) and table 1 and the IT model (figures 6-8) and table 2.

*Figure 3, SD model integration of chronic (T1-T5) and acute oral data (ao1-ao5) with a k_sr_ value of 0.5. The top row gives the exposure profiles, the middle row the scaled damage and the bottom row presents the modelled (line with confidence interval) and observed (dots) survival probability*

*Figure 4, SD model integration of chronic (T1-T5) and acute oral data (ao1-ao5) with a k_sr_ value of 0.625. The top row gives the exposure profiles, the middle row the scaled damage and the bottom row presents the modelled (line with confidence interval) and observed (dots) survival probability*

*Figure 5, SD model integration of chronic (T1-T5) and acute oral data (ao1-ao5) with a k_sr_ value of 1.25. The top row gives the exposure profiles, the middle row the scaled damage and the bottom row presents the modelled (line with confidence interval) and observed (dots) survival probability*

*Figure 6, IT model integration of chronic (T1-T5) and acute oral data (ao1-ao5) with a k_sr_ value of 0.5. The top row gives the exposure profiles, the middle row the scaled damage and the bottom row presents the modelled (line with confidence interval) and observed (dots) survival probability*

*Figure 7, It model integration of chronic (T1-T5) and acute oral data (ao1-ao5) with a k_sr_ value of 0.625. The top row gives the exposure profiles, the middle row the scaled damage and the bottom row presents the modelled (line with confidence interval) and observed (dots) survival probability*

*Figure 8, IT model integration of chronic (T1-T5) and acute oral data (ao1-ao5) with a k_sr_ value of 1.25. The top row gives the exposure profiles, the middle row the scaled damage and the bottom row presents the modelled (line with confidence interval) and observed (dots) survival probability*

Table 1 R^2^, NMRSE and parameter values obtained with the SD model

| *k_sr_*  d^-1^ | R^2^ | NMRSE  % | *k_d_*  d^-1^ | *m_w_*  µg/bee | *b_w_*  (µg/bee d)^-1^ |
| --- | --- | --- | --- | --- | --- |
| 0.5 | 0.940 | 8.14 | 2.8 (1.7–5.3) | 0.96 (0.72-1.9) | 0.034 (0.029-0.045) |
| 0.625 | 0.935 | 8.37 | 2.8 (1.7–5.2) | 0.97 (0.74-2.0) | 0.035 (0.029-0.049) |
| 1.25 | 0.924 | 8.76 | 2.3 (1.6-3.5) | 1.9 (0.92-2.3) | 0.050 (0.036-0.060) |

Table 2 R^2^, NMRSE and parameter values obtained with the IT model

| *k_sr_*  d^-1^ | R^2^ | NMRSE  % | *k_d_*  d^-1^ | *m_w_*  µg/bee | F_s_ |
| --- | --- | --- | --- | --- | --- |
| 0.5 | 0.942 | 6.16 | < 0.056 | 0.38 (0.046-1.5) | 5.5 (4.5-6.9) |
| 0.625 | 0.934 | 6.36 | < 0.063 | 0.56 (0.045-1.6) | 5.4 (4.4-6.8) |
| 1.25 | 0.885 | 8.09 | < 0.079 | 0.93 (0.044-1.8) | 5.2 (4.3-6.5) |

It can be concluded that the model output is not sensitive for value of *k_sr_*. All avlues are well within the confidence intervals for different values of *k_sr_*. The differences in goodness of fit and parameter values for the different *k_sr_* values are marginal for the SD model, where the IT model performs poorer at higher values for *k_sr_*.

Based on physiological considerations, the lower values appear to be more realistic, the actual value for *k_sr_* depends on the feeding condition before the start of the experiment. The feeding condition of the selected bees will be good and therefore the honey stomach of the selected bees is unlikely to be completely empty after the 2 hour starvation period. And after the 2 hour starvation period the bees are able to accommodate the 20 µL of contaminated food without problems, therefore the default setting (with the honey stomach volume not at its maximum) appears to be a realistic value.

### 2.2.2 Contact uptake availability rate constant

In an acute contact experiment a droplet containing the pesticide is dropped in ‘neck’ of the bee ^10^. After that the observation period starts where effects are observed for different initial concentrations. To enhance uptake of the compound by the honey bee that compound of interest is dissolved in a carrier solution, usually acetone. Data on the uptake and elimination rates for acute contact exposure with the use of carrier are scarce. Zaworra et al^8^ published data on uptake- and elimination kinetics based on whole body residues for radio labelled imidacloprid, thiacloprid and Acetamiprid ^8^. In this experiment a carrier (acetone) was used to deliver the pesticide topically to honey bees. These results show that an acute contact exposure experiment cannot be interpreted as a constant exposure over time (48 hrs) but should be treated as a decreasing exposure concentration on the bee over time. A first order decrease of the concentration on the bee fits well with the data presented by Zaworra, and from the plots in this paper contact availability rate constants (*k_ca_*) around 0.5 d^-1^ could be derived.

These values are in line with results obtained by Hillier et al ^11^ who measured total residues in honey bees after dermal application of miticides amitraz and tau-fluvalinate. Other reported values in different species are 50% daily uptake for a radio labelled growth regulator from the benzoylphenylurea group in *Mamestra brassicae* ^12^ and a 75% decline in topically applied Pyrethrin I for the cockroach ^13^.

Like with the honey stomach release rate some different values are tested to investigate the sensitivity of the model for this parameter. The range covering the values derived from literature for *k_ca_* of 0.4, 0.8 and 1.2 d^-1^ rate were used for integration of acute and chronic data and evaluated, both with the SD model (figures 9-11) and table 3 and the IT model (figures 12-14) and table 4. Again Thiacloprid was used as an example.

*Figure 9, SD model integration of chronic (T1-T5) and acute contact data (ac1-ac5) with a k_ca_ value of 0.40. The top row gives the exposure profiles, the middle row the scaled damage and the bottom row presents the modelled (line with confidence interval) and observed (dots) survival probability*

*Figure 10, SD model integration of chronic (T1-T5) and acute contact data (ac1-ac5) with a k_ca_ value of 0.80. The top row gives the exposure profiles, the middle row the scaled damage and the bottom row presents the modelled (line with confidence interval) and observed (dots) survival probability*

*Figure 11, SD model integration of chronic (T1-T5) and acute contact data (ac1-ac5) with a k_ca_ value of 1.20. The top row gives the exposure profiles, the middle row the scaled damage and the bottom row presents the modelled (line with confidence interval) and observed (dots) survival probability*

*Figure 12, IT model integration of chronic (T1-T5) and acute contact data (ac1-ac5) with a k_ca_ value of 0.40. The top row gives the exposure profiles, the middle row the scaled damage and the bottom row presents the modelled (line with confidence interval) and observed (dots) survival probability*

*Figure 13, IT model integration of chronic (T1-T5) and acute contact data (ac1-ac5) with a k_ca_ value of 0.80. The top row gives the exposure profiles, the middle row the scaled damage and the bottom row presents the modelled (line with confidence interval) and observed (dots) survival probability*

*Figure 14, IT model integration of chronic (T1-T5) and acute contact data (ac1-ac5) with a k_ca_ value of 1.20. The top row gives the exposure profiles, the middle row the scaled damage and the bottom row presents the modelled (line with confidence interval) and observed (dots) survival probability*

Table 3 R^2^, NMRSE and parameter values obtained with the SD model

| *k_ca_*  d^-1^ | R^2^ | NMRSE  % | *k_d_*  d^-1^ | *m_w_*  µg/bee | *b_w_*  (µg/bee d)^-1^ |
| --- | --- | --- | --- | --- | --- |
| 0.4 | 0.931 | 5.7 | 0.34 (0.20-0.55) | 0.63 (0.36-1.0) | 0.041 (0.030-0.060) |
| 0.8 | 0.928 | 5.8 | 0.41 (0.26-0.66) | 0.74 (0.42-1.1) | 0.041 (0.030-0.061) |
| 1.2 | 0.923 | 6.1 | 0.49 (0.30-0.76) | 0.80 (0.47-1.3) | 0.040 (0.031-0.060) |

Table 4 R^2^, NMRSE and parameter values obtained with the IT model

| *k_ca_*  d^-1^ | R^2^ | NMRSE  % | *k_d_*  d^-1^ | *m_w_*  µg/bee | F_s_ |
| --- | --- | --- | --- | --- | --- |
| 0.4 | 0.834 | 8.6 | < 0.0078 | 0.075 (0.067-0.35) | 7.9 (6.4 – 10) |
| 0.8 | 0.857 | 7.2 | < 0.0082 | 0.068 (0.061-0.34) | 6.9 (5.6-8.6) |
| 1.2 | 0.867 | 6.4 | < 0.0083 | 0.063 (0.057-0.32) | 6.2 (5.1-7.7) |

It can be concluded that the model performance is not sensitive for value of *k_ca_*. The differences in parameter values for the different *k_ca_* values are well within the confidence intervals for both models, though there is a slight upward trend for *k_d_* and *m_w_* with increasing values for *k_ca_*. The (marginally) better fits in the SD model are obtained for lower values of *k_ca_*, whereas the IT model has a (marginally) better performance with higher values for *k_ca_*.

A visual fit of the data shows that the model fits for the acute data solely shows that the lower value has a better predictive outcome. Therefore the a *k_ca_* value of 0.4 d^-1^ was chosen as the default setting.

### 2.2.3 Comparison between acute oral and acute contact test results

The default values for *k_sr_* and *k_ca_* are comparable and also lead to comparable parameter estimates and therefore also to comparable predictions for the acute oral and acute contact *LD_50_*s. Bayo et al^14^ listed acute oral and acute contact *LD_50_*s, taken from different databases. These results were plotted in the figure below and indeed acute contact and acute oral *LD_50_*s are comparable for a wide range of pesticides, indicating that oral and contact uptake with a carrier solvent must be comparable and so giving a confirmation of the assumptions set out in the previous paragraphs.

Figure 15: 48 hr oral *LD_50_* vs 48 hr contact *LD_50_* for different pesticides containing acaricides, herbicides, fungicides and insecticides

# 3 Model Validation

Validation data are used to test the model performance for predictions of mortality under exposure profiles which have not been used for model calibration. Ideally, effect data from the experiments with time-variable exposure are available for the evaluation of TKTD models. The following checklist is mandatory for invertebrates according the EFSA opinion on TKTD modelling ^1^:

- Are effect data available from experiments under time-variable exposure?
- Is mortality reported at least for 7 time-points in the validation data set?
- Are two exposure profiles tested with at least two pulses each, separated by no-exposure intervals of different duration length?

Effect data under time-variable exposures that were not used for model calibration are to be used for the validation of the model. Therefore the acute oral and an acute contact tests, are used for model validation. With this approach the validation criteria, at least 7 observations over time and a repeated pulse experiment, are not fully met. But the time variability of the validation data is considered the most important criterion, which is met with two independent tests.

The calibrated model output is used for validation with the available time-variable exposures. Five complete and consistent datasets were available for:

- Beta-cyfluthrin
- Deltamethrin
- Dimethoate
- Ethiprole
- Thiacloprid

Validation of the model output was tested for these compounds.

## 3.1 Beta-cyfluthrin

For beta-cyfluthrin several datasets for acute test results were available. Test M-363013 was chosen as this test has observations for the acute contact test up till 4 days where the other tests have observations up till 2 days, so two additional observation over time are available. In addition this test was carried out with 6 concentration levels instead of 5.

SD model

*Fig 1 SD model validation, predictions for acute oral (ao1 – ao6) and acute contact data (ac1-ac6). The top row gives the exposure profiles, the middle row the scaled damage and the bottom row presents the modelled (line with confidence interval) and observed (dots) survival probability*

Model efficiency (NSE, r-square) : 0.8100

Normalised root-means-square error (NRMSE) : 12.6 %

Survival probability prediction error (SPPE) max (ac4) : 29.5 %

Survival probability prediction error (SPPE) min (ao4) : -29.6 %

IT

*Fig 2 IT model validation, predictions for acute oral (ao1 – ao6) and acute contact data (ac1-ac6). The top row gives the exposure profiles, the middle row the scaled damage and the bottom row presents the modelled (line with confidence interval) and observed (dots) survival probability*

Model efficiency (NSE, r-square) : 0.799

Normalised root-means-square error (NRMSE) : 12.9 %

Survival probability prediction error (SPPE) max (AC3) : 24.3 %

Survival probability prediction error (SPPE) min (ao5) : -29.4 %

Both the SD and the IT model give comparable results, which are well within the requirements from the EFSA SO.

## 3.2 Deltamethrin

SD model

*Fig 3 SD model validation, predictions for acute oral (ao1 – ao6) and acute contact data (ac1-ac6). The top row gives the exposure profiles, the middle row the scaled damage and the bottom row presents the modelled (line with confidence interval) and observed (dots) survival probability*

Model efficiency (NSE, r-square) : 0.914

Normalised root-means-square error (NRMSE) : 10.2 %

Survival probability prediction error (SPPE) max (ao2) : 8.8 %

Survival probability prediction error (SPPE) min (ac5) : -15.5%

IT model

*Fig 4 IT model validation, predictions for acute oral (ao1 – ao6) and acute contact data (ac1-ac6). The top row gives the exposure profiles, the middle row the scaled damage and the bottom row presents the modelled (line with confidence interval) and observed (dots) survival probability*

Model efficiency (NSE, r-square) : 0.897

Normalised root-means-square error (NRMSE) : 11.2 %

Survival probability prediction error (SPPE) max (ao1) : +9.7 %

Survival probability prediction error (SPPE) min (ao4) : -18.2 %

Both the SD and the IT model give comparable results, which are well within the requirements from the EFSA SO.

## 3.3 Dimethoate

Dimethoate was used as a positive control in a number of available studies, typically in a limited number of concentrations. The result of the study on Cyanamid (report nr M-001004) was used as a validation dataset. This dataset has 6 tested concentrations, where the majority of the tests has 4 or 5 tested concentrations

SD model

*Figure 5 SD model validation, predictions for acute oral (ao1 – ao6) and acute contact data (ac1-ac6). The top row gives the exposure profiles, the middle row the scaled damage and the bottom row presents the modelled (line with confidence interval) and observed (dots) survival probability*

Model efficiency (NSE, r-square) : 0.8872

Normalised root-means-square error (NRMSE) : 20.9 %

Survival probability prediction error (SPPE) max (ac5) : 28.3 %

Survival probability prediction error (SPPE) min (ao3) : -22.3%

IT model

*Figure 6 IT model validation, predictions for acute oral (ao1 – ao6) and acute contact data (ac1-ac6). The top row gives the exposure profiles, the middle row the scaled damage and the bottom row presents the modelled (line with confidence interval) and observed (dots) survival probability*

Model efficiency (NSE, r-square) : 0.881

Normalised root-means-square error (NRMSE) : 21.5 %

Survival probability prediction error (SPPE) max (ac5) : +27.1 %

Survival probability prediction error (SPPE) min (ao3) : -22.3 %

Both the SD and the IT model give comparable results, which are well within the requirements from the EFSA SO, except for the minimum value of SPPE, which is outside the EFSA requirements

## 3.4 Ethiprole

SD model

*Figure 7 SD model validation, predictions for acute oral (ao1 – ao7) and acute contact data (ac1-ac6). The top row gives the exposure profiles, the middle row the scaled damage and the bottom row presents the modelled (line with confidence interval) and observed (dots) survival probability*

Model efficiency (NSE, r-square) : 0.646

Normalised root-means-square error (NRMSE) : 27.0 %

Survival probability prediction error (SPPE) max (ao4) : 29.7 %

Survival probability prediction error (SPPE) min (ac5) : 38.8 %

IT model

*Fig 8 IT model validation, predictions for acute oral (ao1 – ao7) and acute contact data (ac1-ac6). The top row gives the exposure profiles, the middle row the scaled damage and the bottom row presents the modelled (line with confidence interval) and observed (dots) survival probability*

Model efficiency (NSE, r-square) : 0.661

Normalised root-means-square error (NRMSE) : 26.4 %

Survival probability prediction error (SPPE) max (ao5) : 28.9 %

Survival probability prediction error (SPPE) min (ao5) : -37.2 %

Both the SD and the IT model give comparable results, which are well within the requirements from the EFSA SO.

## 3.5 Thiacloprid

SD model

*Fig 9 SD model validation, predictions for acute oral (ao1 – ao5) and acute contact data (ac1-ac5). The top row gives the exposure profiles, the middle row the scaled damage and the bottom row presents the modelled (line with confidence interval) and observed (dots) survival probability*

Model efficiency (NSE, r-square) : 0.791

Normalised root-means-square error (NRMSE) : 18.2 %

Survival probability prediction error (SPPE) max (ao2) : 17.3 %

Survival probability prediction error (SPPE) min (ac2) : 29.6 %

IT model

*Fig 10 IT model validation, predictions for acute oral (ao1 – ao5) and acute contact data (ac1-ac5). The top row gives the exposure profiles, the middle row the scaled damage and the bottom row presents the modelled (line with confidence interval) and observed (dots) survival probability*

Model efficiency (NSE, r-square) : 0.760

Normalised root-means-square error (NRMSE) : 19.6 %

Survival probability prediction error (SPPE) max (ac2) : 13.9 %

Survival probability prediction error (SPPE) min (ao2) : -35.7 %

Both the SD and the IT model give comparable results, which are well within the requirements from the EFSA SO.

## 3.6 Conclusion on validation

The validation of the model can be evaluated as not complete. Validation data with time-variable exposures were available but not with at least 7 observations over time as is advised by the EFSA SO. In addition a repeated pulse experiment was not available for any of the compounds.

The model was validated with the existing time variable exposure data for all compounds with a complete and consistent dataset. The validation results show that for all compounds the validation requirements set out in the EFSA SO are met.

The overall response pattern in model output and the data is consistent over time and over concentrations. The visual match of the model output and the data is generally good and the NMRSE and SPPE values are well within the ranges of acceptability as laid out in the EFSA SO. The PPC criterion for goodness of fit is not available in the open GUTS modelling framework and was therefore not evaluated.

Overall, the documentation of the GUTS model and its application to predict the survival of honey bees

under exposure to time-variable profiles of different pesticides does comply with the requirements as

announced in the EFSA SO. It is concluded that the quality of the data used has been considered and documented to be of sufficient quality for calibration and validation of the model. The chronic toxicity studies for the honey bees were used for the model calibration. The model documentation and parameter estimation method were evaluated positive and the choice of the data set for model validation was evaluated positive.

Plots of the calibrated GUTS models in comparison with the calibration data over time are provided, and can be considered (visual match, NMRSE values, SPPE values) of good quality. The validation of the model can be evaluated as not complete. Datasets with time-varying exposure concentrations are available and for these datasets parameter values have been estimated and calibrated including confidence intervals, and optimal values were reported and can be considered (visual match, NMRSE values, SPPE values) of good quality. However the validation data generally have 4-5 observations over time and a repeated pulse experiment was not available for any of the compounds.

An analysis of uncertainty in the model output was not performed as such since the uncertainty in the parameter has not been propagated to the model output.

# 4 Fits for compounds with consistent and complete datasets

In this section the validated model is used for the interpretation for compounds with a complete and consistent dataset. A complete dataset implies that there is a chronic test, and acute oral test and an acute contact test available. Consistency implies that if there is more than 1 acute oral or acute contact test available, the test results expressed as 48 hr *LD_50_* are within a factor of 10 (see main text).

Complete and consistent datasets were available for Beta-cyfluthrin, Deltamethrin, Dimethoate, Ethiprole, Imidacloprid and Thiacloprid.

## 4.1 Beta-cyfluthrin

Beta-cyfluthrin has 3 acute test results with considerable differences. The acute oral test with an LD50 of 130 ng/bee fits really well with the effects of the chronic test (if the chronic test is fitted with the acute oral test this gives and R2 value of 0.956). The third test with a reported LD50 of 50 ng/bee fits reasonable well with the chronic test although the predicted effects are somewhat underestimated by the model and finally the second test with an LD50 of 16 ng/bee is out of line with the results of the chronic test but this test also had some dosing issues in the higher concentration ranges. The test with the highest value for the contact exposure (48 LD50 of 38 ng/bee) gives a reasonable fit with the chronic test and therefore as should be expected the effects of the other two tests with a resp 48 hr LD50 of 6 and 12 ng/bee are underestimated by the model.

### 4.1.2 SD model fits

Parameters chronic test 479053 (R^2^ 0.970)

k_d_: 0.99 (0.73 - 1.4) 1/d

m_w_: 9.7 (8.3 - 10.7) [ng/bee/day]

h_b_: 7.1E-03 (4.7E-03 - 0.010) 1/d

b_w_: 9.1E-03 (7.6E-03 - 0.011) 1/(ng/bee d)

*Figure 1 Beta-cyfluthrin chronic test simulation with 5 treatments (T1-T5) and a control. The top panels give the exposure profile. The middle panels show the scaled damage and the bottom panels the fitted survival probability against the observed survival data.*

Beta-cyfluthrin chronic combined with acute test results 51896

kd: 19 (8.4 - 276) 1/d

mw: 5.5e-05 (5.5e-05* - 0.47) ng/bee

hb: 0.0094 (0.0054 - 0.014) 1/d

bw: 0.0048 (0.0042 - 0.0053) 1/(ng/bee d)

*Figure 2 Beta-cyfluthrin chronic test simulation with 5 treatments (T1-T5) and a control combined with the acute oral (oa1 – ao5) and acute contact (ac1 - ac5) test from test nr 51896. The top panels give the exposure profile. The middle panels show the scaled damage and the bottom panels the fitted survival probability against the observed survival data.*

Here it shows that the results of the acute oral test is in line with the chronic test but the effects of the acute contact test are underestimated. If only the acute oral test is combined with the chronic test, this gives an R^2^ value of 0.956).

Beta-cyfluthrin combined with acute test results 36013 R2 0.705

*Figure 3 Beta-cyfluthrin chronic test simulation with 5 treatments (T1-T5) and a control combined with the acute oral (oa1 – ao6) and acute contact (ac1 – ac6) test from test nr 36013. The top panels give the exposure profile. The middle panels show the scaled damage and the bottom panels the fitted survival probability against the observed survival data.*

Here the effects in the higher concentration levels of the acute oral test are underestimated. The two highest test results showed some problems with the dosing as the nominal and actual concentration levels were not in agreement, where the differences in the lower concentration ranges are negligible. Effects in the acute contact test are reasonably in line with the model predictions.

Beta-Cyfluthrin combined with acute test results 53813 R2 0.707

kd: 16 (8.3 - 56) 1/d

mw: 5.8 (4.4 - 6.6) ng/bee

hb: 0.0037 (0.0015 - 0.0068) 1/d

bw: 0.0088 (0.0078 - 0.0097) 1/(ng/bee d)

*Figure 4 Beta-cyfluthrin chronic test simulation with 5 treatments (T1-T5) and a control combined with the acute oral (oa1 – ao6) and acute contact (ac1 – ac6) test from test nr 53813. The top panels give the exposure profile. The middle panels show the scaled damage and the bottom panels the fitted survival probability against the observed survival data.*

Here the results of the acute oral test are in line with the chronic test and especially the medium range concentrations in the acute contact test are underestimated. The effects in the lower and higher concentration ranges are a reasonable agreement with the chronic test results.

### 4.1.2 IT model fits

IT model beta-cyfluthrin cronic R2 0.976

kd: 0.11 (0.060 - 0.16) 1/d

mw: 15 (9.0 - 20) [ug/bee/day]

hb: 6.5E-03 (3.7E-03 - 0.010) 1/d

Fs: 3.5 (2.9 - 4.4) [-]

*Figure 5 Beta-cyfluthrin chronic test simulation with 5 treatments (T1-T5) and a control. The top panels give the exposure profile. The middle panels show the scaled damage and the bottom panels the fitted survival probability against the observed survival data.*

IT model beta-cyfluthrin chronic R2 0.575 (chronic + 51896)

kd: 0.23 (0.16 - 0.28) 1/d

mw: 27 (21 - 31) ng/bee

hb: 0.035 (0.030 - 0.041) 1/d

Fs: 2.9 ( 2.402 - 3.761 ) [-]

*Figure 6 Beta-cyfluthrin chronic test simulation with 5 treatments (T1-T5) and a control combined with the acute oral (oa1 – ao5) and acute contact (ac1 - ac5) test from test nr 51896. The top panels give the exposure profile. The middle panels show the scaled damage and the bottom panels the fitted survival probability against the observed survival data.*

IT model beta-cyfluthrin cronic R2 0.685 (chronic + 36013)

kd: 0.15 (0.10 - 0.20) 1/d

mw: 13 (9.3 - 16) ng/bee

hb: 0.0020 ( 0.00063 - 0.0045) 1/d

Fs: 11 (8.9 - 14) [-]

*Figure 7 Beta-cyfluthrin chronic test simulation with 5 treatments (T1-T5) and a control combined with the acute oral (oa1 – ao6) and acute contact (ac1 – ac6) test from test nr 36013. The top panels give the exposure profile. The middle panels show the scaled damage and the bottom panels the fitted survival probability against the observed survival data.*

IT model beta-cyfluthrin cronic R2 0.713 (chronic + 53813)

kd: 0.19 (0.14 - 0.24) 1/d

mw: 14 (11 - 16) ng/bee

hb: 0.0017 (0.00043 - 0.0041) 1/d

Fs: 9.6 (7.8 - 12) [-]

*Figure 8 Beta-cyfluthrin chronic test simulation with 5 treatments (T1-T5) and a control combined with the acute oral (oa1 – ao6) and acute contact (ac1 – ac6) test from test nr 53813. The top panels give the exposure profile. The middle panels show the scaled damage and the bottom panels the fitted survival probability against the observed survival data.*

## 4.2 Deltamethrin

Deltamethrin has 2 acute oral and two acute contact tests. The contact tests show very similar results but the two oral tests differ by a factor of 7. One of the oral tests gives very poor fits the second oral test gives very good fits in combination with the chronic and acute contact test.

The chronic test had differences in feeding rates in the different concentration levels. The plots and parameter values are based on the actual feeding rates.

### 4.2.1 SD model fits

Parameter values chronic (477250) R^2^ 0.952

kd: 4.4 (3.3 - 7.1) 1/d

mw: 600 (569 - 621) ng/bee

hb: 0.0017 (0.00043 - 0.0044) 1/d)

be: 0.0026 (0.0019 - 0.0035) 1/(ng/bee d)

*Figure 9 Deltamethrin chronic test simulation with 5 treatments (T1-T5) and a control. The top panels give the exposure profile. The middle panels show the scaled damage and the bottom panels the fitted survival probability against the observed survival data.*

In the chronic test the feeding rates have had an effect on the dose-response curves, especially in higher concentrations. Apparently deltamethrin has a repellent effect. The rather strange survival curve in the third treatment where survival drops rather steep in the first few days and then remains almost stable at a consistent level is also not in line with expectations. Overall though the fit is still rather good with an R^2^ value of 0.952.

Parameter values combined (R^2^ 0.558)

kd: <144 1/d

mw: 67 (53 - 121) ng/bee

hb: 1.1E-03 (7.8e-05 – 6.4E-03) 1/d

bw: 7.6E-04 (6.9E-04 – 8.9E-04) 1/(ng/bee d)

The estimate of the dominant rate constant was not really possible for the complete dataset. This was caused by the somewhat poor dose-response curves and the poor fit of the acute contact data.

*Figure 10 Deltamethrin chronic test simulation with 5 treatments (T1-T5) and a control combined with the acute oral (oa1 – ao5 test 149196) and acute contact (ac1 – ac5 test 149494). The top panels give the exposure profile. The middle panels show the scaled damage and the bottom panels the fitted survival probability against the observed survival data.*

The figure shows that the results of the acute oral treatments are rather well described by the model for all concentration levels. But the model predictions of the effects of the acute contact data are underestimated.

When the acute contact data are excluded the predictions become much better R2 0.928

kd: 4.8 (3.6 - 7.7) 1/d

mw: 563 (523 - 594) ng/bee

hb: 0.0017 (0.00042 - 0.0043) 1/d

be: 0.0017 (0.0014 - 0.0021) 1/(ng/bee d)

*Figure 11. Deltamethrin combining chronic with acute oral. The chronic treatments indicated as T1 – T5 (based on 477250), acute oral treatments indicated as ao1 – ao5 (based on 149196). The top panels give the exposure profile. The middle panels show the scaled damage and the bottom panels the fitted survival probability against the observed survival data.*

The second acute test (44971) has a 48 hr LD50 for the acute oral test, which is a factor of 7 lower and an even lower value for the contact test gives very poor fits (R2 = 0.44, figure not shown) with an underestimation of the effects as might be expected (if good fits are obtained for an acute 48 hr oral LD50 of 1.41 ug/bee it is to be expected that a value of 0.2 will lead to an underestimation of the actual effects).

### 4.2.2 IT model fits

It model deltamethrin chronic R2 0.920

kd: 0.72 (0.56 - 0.89) 1/d

mw: 556 (476 - 630) ng/bee

hb: 0.0016 (0.00040 - 0.0043) 1/d

Fs: 2.2 (1.9 - 2.8) [-]

*Figure 12 Deltamethrin chronic test simulation with 5 treatments (T1-T5) and a control. The top panels give the exposure profile. The middle panels show the scaled damage and the bottom panels the fitted survival probability against the observed survival data.*

It model deltamethrin all data R2 0.619

kd: 0.39 (0.32 - 0.47) 1/d

mw: 208 (175 - 245 ) ng/bee

hb: 0.00072 ( 5.2e-05 - 0.0031) 1/d

Fs: 12 (8.9 - 17) [-]

*Figure 13 Deltamethrin chronic test simulation with 5 treatments (T1-T5) and a control combined with the acute oral (oa1 – ao5 test 149196) and acute contact (ac1 – ac5 test 149494). The top panels give the exposure profile. The middle panels show the scaled damage and the bottom panels the fitted survival probability against the observed survival data.*

Deltamethrin chronic + acute oral R2 0.928

kd: 0.62 (0.51 - 0.73) 1/d

mw: 524 (457 - 584) ng/bee

hb: 0.0015 ( 0.00030 - 0.0040) 1/d

Fs: 2.6 (2.3 - 3.1) [-]

*Figure 14 Deltamethrin combining chronic with acute oral. The chronic treatments indicated as T1 – T5 (based on 477250), acute oral treatments indicated as ao1 – ao5 (based on 149196). The top panels give the exposure profile. The middle panels show the scaled damage and the bottom panels the fitted survival probability against the observed survival data.*

Deltamethrin acute 44971 + chronic R2 0.889

kd: 0.41 (0.34 - 0.50 ) 1/d

mw: 430.5 (375 - 496) ng/bee

hb: 0.0007276 (5.1e-05 - 0.0029) 1/d

Fs: 4.3 (3.6 – 5.3) [-]

*Figure 15 Deltamethrin combining chronic with acute oral. The chronic treatments indicated as T1 – T5 (based on 477250), acute oral treatments indicated as ao1 – ao5 (based on 149196). The top panels give the exposure profile. The middle panels show the scaled damage and the bottom panels the fitted survival probability against the observed survival data.*

## 4.3 Dimethoate

The data from the chronic test were taken from a DEFRA report published in the open literature ^15^. The plots in that report were digitalised to obtain the input for the model, which were subsequently used to estimate parameter values and compare the results with acute oral and acute contact data from the available data. All reported LD50s for dimethoate are in a rather close range (see section 1). The results for cyanamide were chosen as the reported LD50s are about the median value of all reported values.

### 4.3.1 SD model fits

Dimethoate chronic exposure R2 0.973

kd: 0.36 (0.28 - 0.44) 1/d

mw: 14 (12 - 15 ) ng/bee

hb: 0.015 (0.010 - 0.020) 1/d

bw: 0.021 (0.018 - 0.028) 1/(ng/bee d)

*Figure 16 Dimethoate chronic test simulation with 5 treatments (T1-T5) and a control. The top panels give the exposure profile. The middle panels show the scaled damage and the bottom panels the fitted survival probability against the observed survival data.*

All data parameter estimates R2 0.800

kd: 0.39 (0.30 - 0.51) 1/d

mw: 13 (12 - 15) ng/bee

hb: 0.014 (0.010 - 0.020) 1/d

bw: 0.014 (0.011 - 0.017) 1/(ng/bee d)

*Figure 17. Dimethoate combining chronic with acute oral and acute contact tests. The chronic treatments indicated as T1 – T5 ^15^, acute oral treatments indicated as ao1 – ao5 (based on 444971) and acute contact treatments indicated as ac1 – ac5 (based on 444971). The top panels give the exposure profile. The middle panels show the scaled damage and the bottom panels the fitted survival probability against the observed survival data.*

The figure shows that both the results of the acute oral and the acute contact test fit in nicely with the chronic test for the whole concentration range.

### 4.3.2 IT model fits

It model dimethoate chronic 0.974

kd: 0.035 (0.0016* - 0.069) 1/d

mw: 6.4 (0.33 - 12) ng/bee

hb: 0.015 (0.0093 - 0.021) 1/d

Fs: 2.2 (2.0 - 2.5) [-]

*Figure 18 Dimethoate chronic test simulation with 5 treatments (T1-T5) and a control. The top panels give the exposure profile. The middle panels show the scaled damage and the bottom panels the fitted survival probability against the observed survival data.*

All data R2 0.829

kd: 0.012 (0.0016* - 0.051) 1/d

mw: 2.4 (0.32 - 9.6) ng/bee

hb: 0.0093 (0.0052 - 0.015) 1/d

Fs: 3.0 (2.7 - 3.4) [-]

*Figure 19 Dimethoate combining chronic with acute oral and acute contact tests. The chronic treatments indicated as T1 – T5 ^15^, acute oral treatments indicated as ao1 – ao5 (based on 444971) and acute contact treatments indicated as ac1 – ac5 (based on 444971). The top panels give the exposure profile. The middle panels show the scaled damage and the bottom panels the fitted survival probability against the observed survival data.*

## 4.4 Ethiprole

### 4.4.1 SD model fits

Parameter estimates chronic R2 0.963

kd: 0.45 (0.26 - 0.77) 1/d

mw: 0.30 (0.22 - 0.34) ng/bee

hb: 1.000e-06 (1.000e-06 - 0.0013) 1/d

bw: 0.31 (0.21 - 0.42) 1/(ng/bee d)

*Figure 20 Ethiprole chronic test simulation with 5 treatments (T1-T5) and a control (test nr 581904). The top panels give the exposure profile. The middle panels show the scaled damage and the bottom panels the fitted survival probability against the observed survival data.*

Parameter estimates Ethiprole chronic and acute combined R2 0.610

kd: 89 (4.4 - 143.8) 1/d

mw: 0.024 (3.8e-05 - 0.18) ng/bee

hb: 1.0e-06 (1.0e-06 - 0.0048) 1/d

bw: 0.036 (0.031 - 0.045) 1/(ng/bee d)

*Figure 21. Ethiprole combining chronic with acute oral and acute contact tests. The chronic treatments indicated as T1 – T5 (test nr 581904), acute oral treatments indicated as ao1 – ao5 (based on 192387) and acute contact treatments indicated as ac1 – ac5 (based on 192387). The top panels give the exposure profile. The middle panels show the scaled damage and the bottom panels the fitted survival probability against the observed survival data.*

It shows that the chronic, the acute oral and the acute contact test can be fitted with good predictive power for the entire concentration ranges.

### 4.4.2 IT model fits

Ethiprole chronic Parameter values R2 0.985

kd: 0.12 (0.040 - 0.19) 1/d

mw: 0.49 (0.22 - 0.65) ng/bee

hb: 1.000e-06 ( 1.000e-06 - 0.001688 ) 1/d

Fs: 2.5 (2.1 - 3.3) [-]

*Figure 22 Ethiprole chronic test simulation with 5 treatments (T1-T5) and a control (test nr 581904). The top panels give the exposure profile. The middle panels show the scaled damage and the bottom panels the fitted survival probability against the observed survival data.*

Parameter estimates chronic and acute combined R2 0.621

kd: 0.0016 (0.0016 - 0.020) 1/d

mw: 0.026 (0.022 - 0.30) ng/bee

hb: 1.0e-06 ( 1.0e-06 - 0.0029) 1/d

Fs: 13 (9.3 - 18) [-]

*Figure 23 Ethiprole combining chronic with acute oral and acute contact tests. The chronic treatments indicated as T1 – T5 (test nr 581904), acute oral treatments indicated as ao1 – ao5 (based on 192387) and acute contact treatments indicated as ac1 – ac5 (based on 192387). The top panels give the exposure profile. The middle panels show the scaled damage and the bottom panels the fitted survival probability against the observed survival data.*

## 4.5 Thiacloprid

### 4.5.1 SD model fits

Parameter estimates SD model R2 0.953

kd: 0.52 (0.23 - 1.3) 1/d

mw: 0.82 (0.43 - 1.4) ug/bee

hb: 0.0045 (0.0025 - 0.0078) 1/d

bw: 0.040 (0.030 - 0.059) 1/(ug/bee d)

*Figure 32 Thiacloprid chronic test simulation with 5 treatments (T1-T5) and a control (test nr 475374). The top panels give the exposure profile. The middle panels show the scaled damage and the bottom panels the fitted survival probability against the observed survival data.*

Thiacloprid acute 001004 combined with chronic R2 0.805

kd: 144 (9.5 - 144) 1/d

mw: 0.92 (0.63 - 1.1) ug/bee

hb: 0.0040 (0.0021 - 0.0066) 1/d

bw: 0.029 (0.026 - 0.033) 1/(ug/bee d)

*Figure 33. Thiacloprid combining chronic with acute oral and acute contact tests. The chronic treatments indicated as T1 – T5 (based on 475374), acute oral treatments indicated as ao1 – ao10 (based on 001004) and acute contact treatments indicated as ac1 – ac6 (based on 001004). The top panels give the exposure profile. The middle panels show the scaled damage and the bottom panels the fitted survival probability against the observed survival data.*

Thiacloprid acute chronic combined acute 000856 R2 0.853

kd: 1.1 (0.73 - 1.7) 1/d

mw: 0.72 (0.45 – 1.0) ug/bee

hb: 0.0040 (0.0021 - 0.0066) 1/d

bw: 0.030 (0.024 - 0.037) 1/(ug/bee d)

*Figure 34. Thiacloprid combining chronic with acute oral and acute contact tests. The chronic treatments indicated as T1 – T5 (based on 475374), acute oral treatments indicated as ao1 – ao5 (based on 000856) and acute contact treatments indicated as ac1 – ac5 (based on 000856).*

It shows the different tests for Thiacloprid can be interpreted within the modelling framework for all treatments, giving good fits for effects in all concentration ranges.

### 4.5.2 IT model fits

IT parameter estimates chronic test R2 0.952

kd: 0.0016 (0.0016 - 0.032) 1/d

mw: 0.055 (0.049 - 0.96) ug/bee

hb: 0.0042 (0.0021 - 0.0070) 1/d

Fs: 5.2 (4.1 - 6.7) [-]

*Figure 35 Thiacloprid chronic test simulation with 5 treatments (T1-T5) and a control (test nr 475374). The top panels give the exposure profile. The middle panels show the scaled damage and the bottom panels the fitted survival probability against the observed survival data.*

Thiacloprid all data acute + chronic study nr 001004 R2 0.780

Kd: 0.033 (0.0016 - 0.081) 1/d

mw: 0.86 (0.044 - 1.9) ug/bee

hb: 0.0028 ( 0.0013 - 0.0051) 1/d

Fs: 8.6 (6.7 - 11) [-]

*Figure 36 Thiacloprid combining chronic with acute oral and acute contact tests. The chronic treatments indicated as T1 – T5 (based on 475374), acute oral treatments indicated as ao1 – ao10 (based on 001004) and acute contact treatments indicated as ac1 – ac6 (based on 001004). The top panels give the exposure profile. The middle panels show the scaled damage and the bottom panels the fitted survival probability against the observed survival data.*

Thiacloprid all data chronic + 000856 combined R2 0.831

kd: 0.0016 (0.0016 - 0.010) 1/d

mw: 0.063 (0.057 - 0.38) ug/bee

hb: 0.0039 (0.0019 - 0.0068) 1/d

Fs: 7.9 (6.6 - 9.6) [-]

*Figure 37 Thiacloprid combining chronic with acute oral and acute contact tests. The chronic treatments indicated as T1 – T5 (based on 475374), acute oral treatments indicated as ao1 – ao5 (based on 000856) and acute contact treatments indicated as ac1 – ac5 (based on 000856).*

# 5 Compounds with inconsistent and complete datasets

In this section the validated model is used for the evaluation of complete and inconsistent datasets. A complete dataset implies that there is a chronic test, and acute oral test and an acute contact test available. Inconsistency implies that there is more than 1 acute oral or acute contact test available and the test results expressed as 48 hr *LD_50_* are larger than a factor of 10 (see main text).

Complete and inconsistent datasets were available for Bromoxynil, Fenamidone, Fenoxaprop, Imidacloprid, Metribuzin, Spiromesifen, Tebuconazole and Tetranilliprole.

First a short evaluation of the data is presented and where possible acute contact, acute oral and chronic tests are integrated and modelled with the BeeGUTS framework with one set of parameter values for the different treatments. The fits for both the SD and the IT model are shown in the sections below.

## 5.1 Bromoxynil

### 5.1.1 SD model

For bromoxynil three acute studies are available; one for bromoxynil octanoate, one for bromoxynil heptanoate and a third for bromoxynil not specified. The chronic study is for bromoxynil also not specified, see Table 5.1.

Table 5.1 Study results for bromoxinyl.

| Compound | BAYER Report nr | 48 h *LD_50_* acute oral  (µg/bee) | 48 h *LD_50_* acute contact  (µg/bee) | 10 d *LC_50_* chronic  (mg/kg) |
| --- | --- | --- | --- | --- |
| Bromoxynil | M-483226 |  |  | 350 |
| Bromoxynil | M-451407 | >201 | >201 |  |
| Bromoxynil | M-444560 | 10.8 | >201 |  |

When the chronic study is used as a starting point this gives a 2 d LD50 of 29..

The fit of the chronic study itself gives an R^2^ value of 0.987 with narrow confidence intervals for the parameter values.

kd 5.6 (2.7 – 7.0) 1/d

mw 29 (25 - 29) ug/bee

hb 0.012 (0.0083 - 0.016) 1/d

bw 1.1 (0.070 - 1.4) 1/( d)

Calculated 2 d LD50 29.4 µg/bee

Based on the calculated *LD_50_* value (assuming a constant exposure) the effect of the acute oral test will be underestimated (reported 48 hr *LD_50_* of 10.8 µg/bee) and the effect of the acute contact test will be overestimated. However the reported 48 hr *LD_50_* of 10.8 µg/bee fits best with the chronic dataset.

## 5.2 Fenamidone

For fenamidone 2 acute tests are available from which one was carried out as a limit test in addition a chronic test was carried out. The limit test does not allow further interpretation and is not taken up in table 5.2.

Table 5.2 Study results for fenamidone.

| Compound | BAYER Report nr | 48 h *LD_50_* acute oral  (µg/bee) | 48 h *LD_50_* acute contact  (µg/bee) | 10 d *LC_50_* chronic  (mg/kg) |
| --- | --- | --- | --- | --- |
| Fenamidone | M-421624 | 57 (72 h) | >93 |  |
| Fenamidone | M-470658 |  |  | 86 |

The acute test consisted of an oral test and a contact test. Since there was less than 50% effect in the highest concentration an *LD_50_* could not be estimated. However for a process-based model this is not an issue, so the results of the acute oral test could be incorporated.

The chronic test could be fitted with an R^2^ value of 0.967 of the available data with narrow confidence intervals.

kd 0.21 (0.032 - 0.48) 1/d

mw 0.32 (0.061 - 0.57) ug/bee

Hb 0.00072 (4.2e-05 - 0.0031) 1/d

bw 0.055 (0.034 - 0.23) 1/(ug/bee d)

The calculated 2d *LD_50_* is 37 ug/bee (assuming a constant exposure).

The acute contact test gives a very good fit over the entire concentration range. In this case the reported 48 hr *LD_50_* of 57 µg/bee fits best with the chronic dataset and is considered to be the most realistic value for the acute test.

## 5.3 Fenoxaprop

The chronic test for fenoxaprop shows very good fit with the data (R2 0.937). But the acute tests were carried out as a limit test, so very limited data is available for the acute test results.

Based on the parameter values of the chronic test a 2 d *LD_50_* was calculated to be 30 µg/bee (assuming a constant exposure). Much lower than the reported 2 d *LD_50_* value of > 100 µg/bee for the contact test and > 109 µg/bee for the oral test.

## 5.4 Imidacloprid

For imidacloprid 5 acute tests are available in addition a chronic test was carried out, the results are shown in table 5.3.

Table 5.3 Study results for fenamidone.

| Compound | BAYER Report nr | 48 h *LD_50_* acute oral  (µg/bee) | 48 h *LD_50_* acute contact  (µg/bee) | 10 d *LC_50_* chronic  (mg/kg) |
| --- | --- | --- | --- | --- |
| Imidacloprid | M-600686 |  |  | 1.31 |
| Imidacloprid | M-006940 | 0.0037 | 0.081 |  |
| Imidacloprid | M-016942 | 0.0409 | - |  |
| Imidacloprid | M-067751 | >0.0347 | 0.0429 |  |
| Imidacloprid | M-067996 | >0.045 | - |  |
| Imidacloprid | M-068023 | >0.0703 | 0.0749 |  |

The chronic test could be fitted with an R^2^ value of 0.971 with narrow confidence intervals.

kd: 3.2 (1.2 - 21) 1/d

mw: 9.8 (3.9 - 14) ng/bee

hb: 0.0020 (1.0e-06 - 0.0051) 1/d

be: 0.032 (0.0066 - 1.1) 1/(ng/bee d)

The calculated 2d *LD_50_* is 24.2 ng/bee (assuming a constant exposure).

This implies that values around 40 ng/bee are most realistic for an acute test, when compared to the chronic test.

A second chronic study was available in the open literature carried out by DEFRA^15^, which shows comparable parameter values to the one that was made available by BAYER and also gave a good fit (R^2^ = 0.946).

kd: 4.9 (1.6 - 144) 1/d

mw: 4.7 (1.9 - 7.9) ng/bee

hb: 0.011 (0.0054 - 0.018) 1/d

bw: 0.0039 (0.0032 - 0.0048) 1/(ng/bee d)

The calculated 2d *LD_50_* from this dataset is 103 ng/bee (assuming a constant exposure).

This implies that the values around 70 ng/bee are most realistic for an acute test, when compared to the chronic test.

## 5.5 Metribuzin

For metribuzin two acute tests were carried out with a large variety in the results but basically only one test (acute oral) with a defined 48 hr *LD_50_* of 34 ug/bee. The contact test (described in the same report) was carried out as a limit test with only concentration (100 µg/bee) with no effects in the highest concentration. The second test was also carried out as a limit test, see table 5.4.

Table 5.4 Study results for metribuzin.

| Compound | BAYER Report nr | 48 h *LD_50_* acute oral  (µg/bee) | 48 h *LD_50_* acute contact  (µg/bee) | 10 d *LC_50_* chronic  (mg/kg) |
| --- | --- | --- | --- | --- |
| Metribuzin | M-540903 |  |  | 620 |
| Metribuzin | M-014115 | >166 | >200 |  |
| Metribuzin | M-294086 | 34 | >100 |  |

The results of the chronic test could be fitted (R^2^ 0.972)

Kd: 0.22 (0.15 - 0.29) 1/d

Mw: 5.1 (4.1 - 5.7) µg/day

Hb: 0.00060 (0.00010 - 0.0026) 1/d

bw: 0.081 (0.057 - 0.11) 1/(µg/day d)

The calculated 2d *LD_50_* from this dataset is 46.4 µg/bee (assuming a constant exposure).

This implies that the measured value of 34 µg/bee is more realistic than the higher reported values.

## 5.6 Spiromesifen

For spiromesifen two acute two acute studies were carried out. The acute contact test has a 2 d *LD_50_* >200 µg/bee. The acute oral tests show a large difference with 2 d *LD_50_* of 60 vs 792 µg/bee resp., see table 5.5.

Table 5.5 Study results for spiromesifen.

| Compound | BAYER Report nr | 48 h *LD_50_* acute oral  (µg/bee) | 48 h *LD_50_* acute contact  (µg/bee) | 10 d *LC_50_* chronic  (mg/kg) |
| --- | --- | --- | --- | --- |
| Spiromesifen | M-657628 |  |  | 9.47 |
| Spiromesifen | M-031874 | 792 | >200 |  |
| Spiromesifen | M-030406 | 60 | >200 |  |

Based on the chronic test result the acute tests are expected to have 2 d *LD_50_*s in the order of 2.5 µg/bee (assuming a constant exposure). This is about two orders of magnitude lower than what was shown in the acute test results. The lowest concentrations in the acute tests (76 µg/bee and 12.5 µg/bee (report nr M-31874 and M-30406 resp) are such that this should kill the bees instantly in the chronic test.

So for spiromesifen the acute data and the chronic data cannot be modelled with one single set of parameter values. This is caused by the specific mode of action. Spiromesifen hampers the processing of food and so effectively it starves the organism. This takes time and therefore the acute test results do not reflect the actual toxicity of this compound. See also the main text.

## 5.7 Tetraniliprole

For tetraniliprole two acute tests were available. The acute oral tests have a reported 48 hr *LD_50_* of 0.11 and 0.01 µg/bee (test nr M-438810 and M-441758 resp), see table 5.6.

Table 5.6 Study results for tetranilliprole.

| Compound | BAYER Report nr | 48 h *LD_50_* acute oral  (µg/bee) | 48 h *LD_50_* acute contact  (µg/bee) | 10 d *LC_50_* chronic  (mg/kg) |
| --- | --- | --- | --- | --- |
| Tetraniliprole | M-438810 | 0.11 | 0.97 |  |
| Tetraniliprole | M-441758 | 0.01 | 1.2 |  |
| Tetraniliprole | M-551955 |  |  | 0.58 |

Based on the chronic test, a 2d *LD_50_* of 0.04 µg/bee was calculated (assuming a constant exposure).

The combination of the chronic test and the acute oral test (report nr M-438810) gives a good fit of the combined exposure routes (R2 0.957), see figure 5.1.

*Figure 5.1 Tetranilliprole combining chronic with acute oral tests. The chronic treatments indicated as T1 – T5, acute oral treatments indicated as ao1 – ao5. The top panels give the exposure profile. The middle panels show the scaled damage and the bottom panels the fitted survival probability against the observed survival data.*

The results of the acute contact tests do not fit with the acute oral tests. In literature test results for tetranilliprole are also available^16^. The reported 10 d *LD_50_* of the chronic test: 0.014 µg/bee, is in line with this research with a value of 0.013 µg/bee, so there is no reason to doubt the results of the chronic test. Therefore acute toxicity values around 0.1 µg/bee are expected for both the acute oral and the acute contact tests. A value of 0.4 µg/bee was mentioned for its 48 hr contact *LD_50_* was mentioned in literature and a range for acute oral exposure is mentioned between 0.1 and 0.5 µg/bee. These values are more in line with the chronic data that are available and therefore are considered to be more realistic.

# 6 References

1. EFSA; Products, Panel o. P. P.; Residues, t.; Ockleford, C.; Adriaanse, P.; Berny, P.; Brock, T.; Duquesne, S.; Grilli, S.; Hernandez-Jerez, A. F.; Bennekou, S. H.; Klein, M.; Kuhl, T.; Laskowski, R.; Machera, K.; Pelkonen, O.; Pieper, S.; Smith, R. H.; Stemmer, M.; Sundh, I.; Tiktak, A.; Topping, C. J.; Wolterink, G.; Cedergreen, N.; Charles, S.; Focks, A.; Reed, M.; Arena, M.; Ippolito, A.; Byers, H.; Teodorovic, I., Scientific Opinion on the state of the art of Toxicokinetic/Toxicodynamic (TKTD) effect models for regulatory risk assessment of pesticides for aquatic organisms. *EFSA Journal* **2018,** *16*, (8), e05377.

2. Ashauer, R.; Albert, C.; Augustine, S.; Cedergreen, N.; Charles, S.; Ducrot, V.; Focks, A.; Gabsi, F.; Gergs, A.; Goussen, B.; Jager, T.; Kramer, N. I.; Nyman, A.-M.; Poulsen, V.; Reichenberger, S.; Schäfer, R. B.; Van den Brink, P. J.; Veltman, K.; Vogel, S.; Zimmer, E. I.; Preuss, T. G., Modelling survival: exposure pattern, species sensitivity and uncertainty. *Scientific Reports* **2016,** *6*, (1), 29178.

3. Jager, T.; Albert, C.; Preuss, T. G.; Ashauer, R., General Unified Threshold Model of Survival - a Toxicokinetic-Toxicodynamic Framework for Ecotoxicology. *Environmental Science & Technology* **2011,** *45*, (7), 2529-2540.

4. Jager, T.; Ashauer, R., How to Evaluate the Quality of Toxicokinetic-Toxicodynamic Models in the Context of Environmental Risk Assessment. *Integrated environmental assessment and management* **2018,** *14*, (5), 604-614.

5. Ashauer, R.; Thorbek, P.; Warinton, J. S.; Wheeler, J. R.; Maund, S., A method to predict and understand fish survival under dynamic chemical stress using standard ecotoxicity data. *Environmental Toxicology and Chemistry* **2013,** *32*, (4), 954-965.

6. OECD, 54 Current approaches in the statistical analysis of ecotoxicity data: A guidance to application. In *54 OECD SERIES ON TESTING AND ASSESSMENT*, OECD, Ed. Paris, 2006.

7. Fournier, A.; Rollin, O.; Le Féon, V.; Decourtye, A.; Henry, M., Crop-Emptying Rate and the Design of Pesticide Risk Assessment Schemes in the Honey Bee and Wild Bees (Hymenoptera: Apidae). *Journal of economic entomology* **2014,** *107*, 38-46.

8. Zaworra, M.; Koehler, H.; Schneider, J.; Lagojda, A.; Nauen, R., Pharmacokinetics of Three Neonicotinoid Insecticides upon Contact Exposure in the Western Honey Bee, Apis mellifera. *Chemical research in toxicology* **2019,** *32*, (1), 35-37.

9. OECD, Test No. 213: Honeybees, Acute Oral Toxicity Test. In 1998.

10. OECD, *Test No. 214: Honeybees, Acute Contact Toxicity Test*. 1998.

11. Hillier, N. K.; Frost, E. H.; Shutler, D., Fate of Dermally Applied Miticides Fluvalinate and Amitraz Within Honey Bee (Hymenoptera: Apidae) Bodies. *Journal of Economic Entomology* **2013,** *106*, (2), 558-565.

12. Tada, M.; Mitsui, T.; Tokuda, K.; Mengel, R.; Wakabayashi, K., Penetration, Absorption and Translocation of 1-(3, 5-Dichloro-2, 4-difluorophenyl)-3-(2, 6-difluorobenzoyl)urea (CME-134) in the Larvae of the Cabbage Armyworm, <i>Mamestra brassicae</i>. *Journal of Pesticide Science* **1987,** *12*, (3), 455-460.

13. Burt, P. E.; Lord, K. A.; Forrest, J. M.; Goodchild, R. E., THE SPREAD OF TOPICALLYâAPPLIED PYRETHRIN I FROM THE CUTICLE TO THE CENTRAL NERVOUS SYSTEM OF THE COCKROACH PERIPLANETA AMERICANA. *Entomologia Experimentalis et Applicata* **1971,** *14*, (3).

14. Sanchez-Bayo, F.; Goka, K., Pesticide residues and bees--a risk assessment. *PloS one* **2014,** *9*, (4), e94482.

15. DEFRA *Assessment of the risk posed to honeybees by systemic pesticides*; DEFRA: 01-03-2007, 2007.

16. authority, A. p. a. v. m., On the evaluation of tetraniliprole in the product Vayego 200 SC insecticide APVMA product nr 86756. In Australian pesticides and veterinary medicines authority: Sydney Australia, 2020.
